# Acute and two-week effects of neotame, stevia rebaudioside M and sucrose-sweetened biscuits on postprandial appetite and endocrine response in adults with overweight/obesity—a randomised crossover trial from the SWEET consortium

**DOI:** 10.1016/j.ebiom.2024.105005

**Published:** 2024-03-28

**Authors:** Catherine Gibbons, Kristine Beaulieu, Eva Almiron-Roig, Santiago Navas-Carretero, J. Alfredo Martínez, Beverley O’Hara, Dominic O’Connor, Julie-Anne Nazare, Alain Le Bail, Cécile Rannou, Charlotte Hardman, Moon Wilton, Louise Kjølbæk, Corey Scott, Hariklia Moshoyiannis, Anne Raben, Joanne A. Harrold, Jason C.G. Halford, Graham Finlayson

**Affiliations:** a School of Psychology, Faculty of Medicine & Health, University of Leeds, UK; b University of Navarra, Faculty of Pharmacy and Nutrition, Dept. of Food Science and Physiology, Center for Nutrition Research, Pamplona, Spain; c Navarra Institute for Health Research (IdiSNa), Pamplona, Spain; d Spanish Biomedical Research Centre in Physiopathology of Obesity and Nutrition (CIBERobn), Institute of Health Carlos III, Madrid, Spain; e Human Nutrition Research Center Rhône-Alpes, Lyon 1 Claude Bernard University, France; f ONIRIS - UMR CNRS GEPEA 6144, Oniris, France; g Department of Psychology, Institute of Population Health, University of Liverpool, UK; h Department of Nutrition, Exercise and Sports, University of Copenhagen, Denmark; i Core Research and Development, Cargill, Inc, USA; j International Reference Laboratory Services, Bioiatriki S.A., Athens, Greece; k Clinical Research, Copenhagen University Hospital – Steno Diabetes Center Copenhagen, Herlev, Denmark

**Keywords:** Sweeteners, Sweetness enhancers, Solid food, Appetite, Glycaemia, Endocrine response

## Abstract

**Background:**

Sweeteners and sweetness enhancers (S&SE) are used to replace energy yielding sugars and maintain sweet taste in a wide range of products, but controversy exists about their effects on appetite and endocrine responses in reduced or no added sugar solid foods. The aim of the current study was to evaluate the acute (1 day) and repeated (two-week daily) ingestive effects of 2 S&SE vs. sucrose formulations of biscuit with fruit filling on appetite and endocrine responses in adults with overweight and obesity.

**Methods:**

In a randomised crossover trial, 53 healthy adults (33 female, 20 male) with overweight/obesity in England and France consumed biscuits with fruit filling containing 1) sucrose, or reformulated with either 2) Stevia Rebaudioside M (StRebM) or 3) Neotame daily during three, two-week intervention periods with a two-week washout. The primary outcome was composite appetite score defined as [desire to eat + hunger + (100 − fullness) + prospective consumption]/4.

**Findings:**

Each formulation elicited a similar reduction in appetite sensations (3-h postprandial net iAUC). Postprandial insulin (2-h iAUC) was lower after Neotame (95% CI (0.093, 0.166); p < 0.001; d = −0.71) and StRebM (95% CI (0.133, 0.205); p < 0.001; d = −1.01) compared to sucrose, and glucose was lower after StRebM (95% CI (0.023, 0.171); p < 0.05; d = −0.39) but not after Neotame (95% CI (−0.007, 0.145); p = 0.074; d = −0.25) compared to sucrose. There were no differences between S&SE or sucrose formulations on ghrelin, glucagon-like peptide 1 or pancreatic polypeptide iAUCs. No clinically meaningful differences between acute vs. two-weeks of daily consumption were found.

**Interpretation:**

In conclusion, biscuits reformulated to replace sugar using StRebM or Neotame showed no differences in appetite or endocrine responses, acutely or after a two-week exposure, but can reduce postprandial insulin and glucose response in adults with overweight or obesity.

**Funding:**

The present study was funded by the 10.13039/501100007601Horizon 2020 program: Sweeteners and sweetness enhancers: Impact on health, obesity, safety and sustainability (acronym: SWEET, grant no: 774293).

**Research in context**

**Evidence before this study**

Reducing sugar consumption has become a key public health target in the fight to reduce the rising burden of obesity-related metabolic diseases such as type 2 diabetes. It is highlighted by Diabetes UK in their position statement on the use of low or no calorie sweeteners, which calls for high quality human trials that examine the effects of non-nutritive sweetener and sweetness enhancers (S&SE) on appetite, food preferences and energy compensation. There is presently a lack of randomised controlled trials that examine specific S&SE on appetite and endocrine responses in solid foods, particularly studying both acute and repeated consumption (two weeks)—these are crucial mechanisms to examine to substantiate the longer-term implications of S&SE use for body weight and blood glucose control.

The current research is especially timely as the use of S&SE has received a lot of negative attention in 2023, including high profile publications linking S&SE consumption with impaired glycaemic response, toxicological damage to DNA and increased risk of heart attack and stroke. All these high profile articles drew a lot of scepticism and debate from scientists following publication. Nevertheless they were reported widely in the media and contribute to the current befuddlement among the general public and especially people at risk of metabolic diseases concerning the safety and supposed benefit of S&SE consumption to reduce sugar in the diet.

Perhaps most notable is the much anticipated WHO guideline on use of S&SE for weight control published in May 2023 which was covered intensely in the mainstream media. The conditional recommendation within this report was that S&SE should *not* be used for weight control. However, the rationale behind the recommendation appears to be founded on a lack of robust evidence (e.g., randomised controlled trials) for a clear long term benefit, coupled with weak evidence from cohort and case–control studies showing an association (not causation) between S&SE intake with higher BMI and poorer health outcomes. In the accompanying review of 283 studies commissioned by the WHO, it was noted that there is a need for highly controlled human nutrition and behaviour research.

**Added value of this study**

In this paper, we present the results of an intensive human clinical trial (randomised crossover with 3 x 2-week intervention periods) investigating the effect of acute and repeated consumption (daily for 2 weeks) of two specific and widely used S&SE—neotame (“artificial”) and stevia rebaudioside M (“plant-based”)—compared to sucrose on appetite-related behavioural, endocrine and health outcomes. The study is important because to date, virtually all studies of the effects of S&SE on appetite and glycaemia have been conducted using *beverages* as the vehicle; few S&SE studies include volunteers with overweight or obesity, or both sexes; most studies only examine a single S&SE (mostly aspartame) compared to a control; and very few studies examine the effect of repeated daily intake of a known S&SE incorporated into the normal diet. These previous limitations and unknowns are addressed in the current manuscript.

As a major work package of the €9 M SWEET project (EU Horizon 2020 grant agreement No 774293), this study consists of a double-blind randomised cross-over trial carried out at 2 sites across 2 European countries. A common solid food matrix was tested across 3 formulations: Sucrose-sweetened control and 2 reformulated products with neotame or StRebM. Participants with overweight and obesity consumed one portion of each formulation daily for 14 days in a fully crossover design. The randomisation strategy used a Latin square design (6 treatment orders) to randomly allocate product sequence into blocks of 6. Each sequence was stratified by sex (female/male) and age group (18–45 years/46–60 years). A female to male ratio of 60/40 within each intervention centre was pre-determined to reflect the target population characteristics. The primary endpoint was composite appetite score while secondary endpoints included food preferences, postprandial glucose and insulin response and other satiety-related peptides (ghrelin, GLP-1 and pancreatic polypeptide). In our data analysis plan we pre-specified both unadjusted and adjusted models performed with formulation, ED and formulation∗ED interaction as fixed factors (including the intercept) and with participant ID number (PPID) and intervention order nested within PPID as random factors. In the adjusted models, intervention site, participant age, sex, BMI, adverse events, and concomitant medication were examined as potential covariates.

**Implications of all the available evidence**

The results of the study indicate no acute or repeated consumption differences between Neotame, StRebM or sucrose on appetite or satiety-related endocrine responses when consumed in a solid food matrix—highlighting that there is no detrimental impact of replacing sugar with S&SE in these endpoints. Additionally, glucose and insulin responses were blunted after acute and repeated consumption of S&SE reformulated biscuits, which may confer a benefit for blood glucose control, for example in individuals at risk of developing type 2 diabetes.

## Introduction

Rates of obesity have risen continually over the last 40 years.^1^ Increased body weight is caused by energy intake exceeding energy expenditure, often facilitated by a diet too rich in available energy.^2^, ^3^, ^4^ Moreover, nutrient-specific models propose that altered hormonal responses to diets high in simple carbohydrates (e.g., sugars) preferentially promote fat storage and weight gain.^2^

Free sugar intake has drawn focus from health professionals and policy makers seeking to influence obesity because of its low nutritional value (lack of vitamins, minerals or fibre), its potential to add to overall energy consumed, facilitating weight gain,^4^ and potentially problematic appetite and endocrine responses to carbohydrates (sugars) relative to other macronutrients.^5^ Simply restricting free sugars from the diet without substitution may reduce diet palatability or contribute to changes in sweet craving,^6^^,^^7^ resulting in poor acceptance and adherence to the diet. The replacement of free sugars with non-nutritive sweeteners and sweetness enhancers (S&SE) in food products is one of the most widely used dietary and food manufacturing strategies to reduce sugar intake and improve the nutritional profile of commercial foods and beverages.

In juxtaposition, a recent World Health Organisation (WHO) guideline^8^ has been published on the use of S&SE for weight control. The conditional recommendation is that S&SE should not be used for weight control or reducing the risk of noncommunicable diseases. However, the rationale behind the recommendation appears to be founded on the lack of robust evidence from randomised controlled trials [RCT] for a clear long term benefit, coupled with weak evidence from cohort and case–control studies for an association between S&SE intake with higher BMI and poorer health outcomes.^9^ Crucially, the mechanistic studies required to substantiate and explain these associations, such as any short term deleterious impacts of S&SE on appetite and endocrine responses, were not part of the remit and therefore are largely ignored. Furthermore, the majority of available evidence on S&SE and health outcomes has examined S&SE consumption in beverages. There is a surprising absence of RCT research on S&SE intake in solid foods, which account for a much greater proportion of energy in the diet.

Since 2018, the use of high intensity S&SE in fine baked goods was prohibited in the European Union after an amendment to Annex II of EC Regulation 1333/2008. Consequently, the use of polyols (mainly maltitol and sorbitol) to replace added sugars in biscuits has become of interest to food manufacturers as a solution to achieve no-added sugar status according to EU regulation on nutrition claims, catering to consumers who wish to control their sugar intake.^10^ Nevertheless, the complete removal of sugars from common solid foods such as baked goods is technically very challenging without having a negative impact on the quality of the product.^11^ Sucrose in a baked product serves several functions, namely, to sweeten, act as a bulking agent, retain moisture, add organoleptic properties, and extend the product shelf-life. These challenges may explain why most human nutrition research on S&SE has utilised beverages as the vehicle of administration and evidence regarding the effects of S&SE in baked goods is limited.^12^

Neotame and Stevia Rebaudioside M (StRebM) are two widely used S&SE in food manufacturing of the 11 currently approved in the EU. While serving the same function in replacing the sweet taste of sugars in foods, these compounds are chemically heterogeneous and derived, absorbed, metabolised, and excreted differently,^13^ illustrating why making comparisons between S&SE is difficult. There is a dearth of studies that specifically investigate the effects of Neotame and StRebM on appetite and metabolic outcomes.^m^ Neotame is a derivative of and chemically similar to Aspartame, but is between 30 and 60 times sweeter, due the fact that it can act on both hydrophobic binding sites of the human sweet receptors at the same time.^14^ StRebM from the Stevia Rebaudiana Bertoni plant is one of more than 60 naturally occurring steviol glycosides with a similar molecular structure.^15^ Whilst StRebA is the most widely used, StRebM is a larger molecule with an additional glucose molecule attached, and is noted as more sweet and less bitter, but there is no reason to suspect that StRebA or StRebM differ metabolically.

One of the reasons for the current partial understanding of the appetitive effects of S&SE in humans is that different S&SE are often assumed to have similar effects on eating behaviour.^12^^,^^16^^,^^17^ However, a 12-week investigation of 4 distinct S&SE reported directionally dissimilar effects of saccharin, aspartame and Stevia Rebaudioside A (StRebA) compared to sucralose on body weight.^18^ A recent review comparing different S&SE suggests that some have the potential to enhance appetite,^12^ although a review into aspartame/acesulfame-K blends found lower energy intake compared to controls but could not attribute this to changes in appetite; with glucose and incretins appearing to be unaffected.^19^ Even fewer studies have compared the effects of specific S&SE on endocrine responses (glycaemic, insulinemic and satiety biomarkers), and those that are available differ in the S&SE used. Stevia has been shown to reduce postprandial glucose compared to sucrose and to reduce insulin compared to aspartame and sucrose,^20^ although it should be noted that the preloads used in this study were not isocaloric. Stevia, compared to maize starch has also been shown to decrease glucagon and glucose (but not Glucagon-like Peptide-1 (GLP-1) or Gastric Inhibitory Peptide (GIP)) in patients with type 2 diabetes.^21^ A recent study comparing aspartame, monk fruit, stevia and sucrose-sweetened beverages consumed before a standardised meal found no difference in glucose and insulin responses over a 3-h period.^22^ More recently, a systematic review and meta-analysis has corroborated these earlier findings that beverages with single S&SE or blends of S&SE had little effect (i.e., act similar to water) on glucose, insulin, GLP-1, GIP, PYY, ghrelin and glucagon.^23^

Given the current controversy surrounding the benefit of S&SE for weight control^24^ the use of S&SE in the food supply increasing in response to consumer demand^25^; and government policies and initiatives to reduce sugar consumption,^26^, ^27^, ^28^, ^29^ there is a pressing need to examine the appetite-related behavioural and endocrine responses of consuming specific S&SE, particularly in solid food matrices. The aim of the current study was therefore to evaluate the acute (1 day) and repeated (two-week daily) ingestive effects of 3 formulations of biscuit with fruit filling on appetite and endocrine responses in adults with overweight and obesity. The formulations developed for this randomised crossover trial contained no added sugar and used either StRebM or Neotame with polyols compared to a sucrose-sweetened control.

## Methods

### Study participants and ethical considerations

**Fig. 1:**
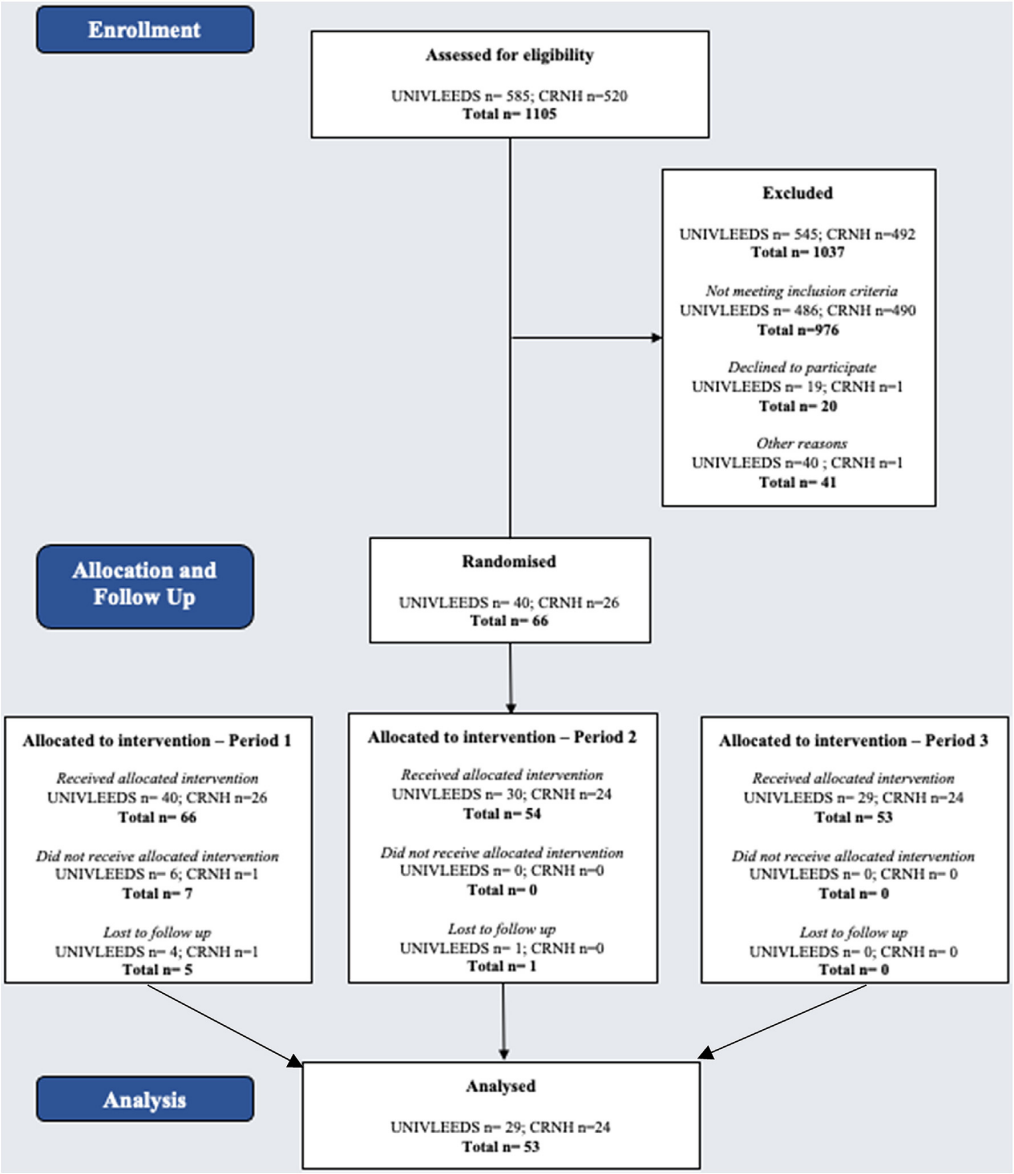
Consort diagram to show the participant flow during the trial.

This study is part of the SWEET project (https://sweetproject.eu/) and reports the outcomes from a two-centre study (University of Leeds, UK and Centre de Recherche en Nutrition Humaine Rhône-Alpes (CRNH), France) conducted between 2021 and 2022. Participants were recruited through posters/leaflets, online advertising and participant research databases. In the UK, ethical approval was granted by the University of Leeds School of Psychology Ethics Committee (PSC-127, approved 19th November 2020) and in France, by the Comité de Protection des Personnes Nord-Ouest III (2021–2042, approved 28th March 2021). Fig. 1 provides details of the participant flow during the trial. All study procedures were conducted in accordance with the Helsinki Declaration and good clinical practice, and the study protocol is registered at ClinicalTrials.gov (NCT04633681). All participants received written and oral information about the study and only trained study personnel were used to provide information, monitor and attest signing of the informed consent form.

### Study protocol

#### Screening and inclusion/exclusion criteria

The full study protocol has been described in Gibbons et al.^30^ In brief, individuals were screened for eligibility including healthy, male and female (self-reported) adults aged 18–60 years, non-smokers, with overweight or obesity (BMI 25–35 kg/m^2^), score <20 on the Eating Attitudes Test (EAT-26),^31^ score ≥3 out of 11 on a short food frequency questionnaire related to habitual sweet product consumption, and rating the control product as ≥40 on a 100-point liking visual analogue scale (VAS) during an initial taste test before the first laboratory session. Anyone currently dieting, or having lost or gained >4.5 kg in the last 3 months were excluded. Food allergies, intolerances, restriction or avoidance of any of the study foods (e.g., veganism) or history of anaphylactic reaction to any food were also excluded. The full list of inclusion and exclusion criteria can be found in.^30^

#### Trial design and randomisation

Participants consumed biscuits containing either Neotame, StRebM or sucrose for 2 weeks in a randomised, crossover, double blind design. Participants attended a laboratory session (exposure day; ED) at the beginning (day 1) and end (day 14) of each consumption period and observed a washout period of 14–21 days in between consumption periods, resulting in all participants completing the three product formulations in a Latin Square design (involving a minimum 70 days study duration plus 7–14 days extension of washout period to aid scheduling). Each randomisation sequence was stratified by sex (female/male) and age group (18–45 years/46–60 years). When feasible, a female/male ratio of minimum 60/40 was also considered to reflect the target population characteristics. One lead researcher (not directly conducting the laboratory measurements) generated the randomisation sequence for each participant. During the at-home intervention periods, participants consumed a portion of the biscuit at a time and place of their choosing using a substitution strategy for similar energy-containing sweet foods in their habitual diet. Compliance with the at-home intervention was assessed via intervention booklets completed daily and by returning the empty food packaging.

#### Food products

Control biscuits with fruit filling (3 units per portion, containing sucrose) were developed to be similar to commercially available produce along with reformulated no added sugar biscuits with fruit filling (containing StRebM or Neotame). Details and nutritional information about the 3 formulations can be seen in Table 1. The reformulation of food products is extremely complex, the development of these biscuits is further explained in,^10^ particularly highlighting the necessity of polyols being used as sugar substitutes. All 3 biscuits were matched for sweetness intensity, flavour and physical appearance, and there was no difference in the perceived pleasantness between the 3 biscuits. The food products were matched in terms of the packaging and were distinguished using a three digit code. Both participants and researchers were blind to the ingredient in the biscuits and unblinding only took place after statistical analysis was complete. St Reb M (95% Steviol Glycosides, 80% Rebaudioside M) as a stevia leaf extract was provided by Cargill (Vilvoorde, BE). Neotame was kindly provided as a gift from ManusBio (Augusta, GA). A photograph of the biscuits can be found in Supplementary Material S1 and full ingredient list in.^30^

**Table 1:** Energy and nutrient composition of the intervention products.

Biscuit	Sucrose Control		S&SE Reformulated	
	Per 100 g	Per portion (3 biscuits)	Per 100 g	Per portion (3 biscuits)
Energy (kcal)	423	360	384	326
Energy (kJ)	1783	1516	1609	1368
Fat (g)	11.2	9.5	11.5	9.8
Sat. fat (g)	7.11	6.0	7.33	6.2
Carbohydrate (g)	75.9	64.5	76.2	64.8
Sugar (g)	24.7	21.0	1.8	1.5
Polyol (g)	3.7	3.1	22.7	19.3
Fibre (g)	0.7	0.6	2.4	2.0
Protein (g)	6.5	5.5	6.6	5.6
Salt (mg)	0.7	0.6	0.7	0.6

#### Exposure day procedure

**Fig. 2:**
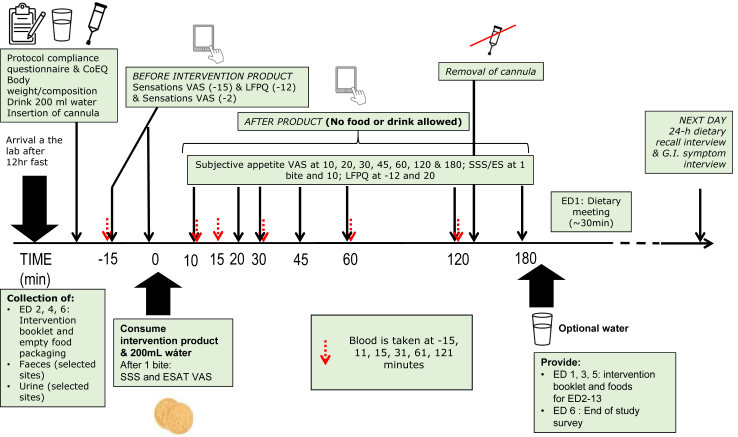
Timeline of events during an Exposure Day. CoEQ, Control of Eating Questionnaire; ED, Exposure Day; ESAT, Expected Satiety; G.I., gastrointestinal; LFPQ, Leeds Food Preference Questionnaire; SSS, Sensory-Specific Satiety; VAS, Visual Analogue Scale.

Participants were instructed to eat a similar evening meal before fasting for a minimum of 12 h prior to attending the laboratory. Exposure day (ED) procedures are represented in Fig. 2. The ED started with a compliance assessment prior to any measurements. Participants then consumed 200 mL of water before an intravenous cannula was inserted into an antecubital vein. The cannula was allowed to rest for 15 min before the fasting sample was taken. Fasting levels of subjective appetite (hunger, fullness, thirst, desire to eat, prospective intake, nausea, bloating, appetite for something savoury and for something sweet, sensory-specific satiety) were then taken on a validated 100-point VAS on a bespoke online questionnaire delivery platform.^32^^,^^33^ Next, food reward—explicit liking and implicit wanting for fatty and sweet foods—was measured using a culturally adapted version of the Leeds Food Preference Questionnaire (LFPQ).^34^ After another VAS measure, participants were served the portion of biscuits (3 units). They were then asked to take one bite of the biscuit and answer questions about expected satiety^35^ and sensory-specific satiety^35^ by VAS. Participants were allowed 10 min to consume the rest of the biscuits followed by serial VAS measures and blood samples at the timepoints noted in Fig. 2. The LFPQ was repeated 20 min post consumption. Once the 180-min postprandial period was complete the participants were offered water and a snack and were free to leave the unit. Glucose and insulin were measured at 0, 10, 15, 30, 60, and 120 min. Ghrelin and glucagon-like peptide 1 (GLP-1) were measured at 0, 30 and 60 min. Pancreatic polypeptide (PP) was measured at 0, 10, 15 and 30 min. Insulin, glucose and PP, were measured to capture the cephalic-phase response to the intervention product (first 30 min after consumption). For further details on methods of analysis for the blood biochemistry measures see.^30^

### Primary and secondary outcomes

This trial has one primary outcome which is the difference in iAUC for the 180-min composite appetite score based on hunger, fullness (reverse scored), desire to eat and prospective food consumption (36) from acute (ED1) compared to repeated exposure (ED14). Secondary outcomes included other subjective sensations (appetite for savoury and sweet, thirst, nausea and bloating), food preferences (liking and wanting for fatty or sweet foods) and glyceamic and endocrine responses (glucose, insulin, ghrelin, GLP-1 and PP) from acute (ED1) compared to repeated exposure (ED14).

### Data processing and statistical analyses

The target sample of 53 was achieved, based on power calculations and published data for the primary outcome,^36^ with an overall sample of 53 completers,^37^ sufficient to detect incremental AUC (niAUC) differences of ∼10% considered to be of practical relevance.^38^ Full information regarding data processing can be found in.^30^ Calculations used for endpoints can be found in Supplementary Material S2. The primary outcome was computed as: = [desire to eat + hunger + (100 − fullness) + prospective consumption]/4.^39^

The trapezoid method^40^ was used to calculate incremental AUC (iAUC) for serum and plasma edocrine paramters (glucose, insulin, ghrelin, GLP-1 and PP) as recommended by Brouns et al.^41^ For appetite sensations the net incremental (niAUC) was used.

Statistical analyses were performed in SPSS version 28 (IBM Corp). Assumptions of regression analysis were conducted including checking normality of the data and influential outliers. Where data were not normally distributed, we log transformed—this was the case for the blood biochemistry data. Outcome variables were visually inspected for normality using histograms and if necessary, data were transformed (e.g., log) to reach normality. Blood biochemistry data was log transformed (glucose, insulin, ghrelin, GLP-1 and PP). Outliers were identified via boxplot and extreme outliers were removed if outside of normal expected range (above 3rd quartile + 3∗interquartile range [IQR] or below 1st quartile—3∗IQR). There were no indications of non-linearity or heteroskedasticity.

Absolute differences in outcomes between ED1 and ED14 were analysed using linear mixed-effects models to compare S&SE product formulations vs. the sucrose control in a 3 (Neotame, StRebM, sucrose control) x 2 (ED1 and ED14) within-subject design. Where appropriate, change score (from ED1 to ED14) analyses were carried out using linear mixed-effects models in a 3 condition (Neotame, StRebM, sucrose control) within-subject design.

Firstly, an unadjusted model was performed with formulation, ED and formulation∗ED interaction as fixed factors (including the intercept) and with participant ID number (PPID) and intervention order nested within PPID as random factors. Next, an adjusted model, including intervention site, participant age, sex, BMI, adverse events, and concomitant medication as covariates was performed. Only covariates that were significant in the model were included in the final adjusted model. Both unadjusted and adjusted models are reported for completeness.

All main analyses were performed blind to allocation. After product formulation codes were unblinded, if a significant main effect of formulation was detected, planned contrasts for each S&SE vs. sucrose were performed.

Following the American Statistical Association’s policy statement on p-values, all p-values from significant effects were reported along with point estimates and confidence intervals to help interpret the compatibility of the data with the study outcomes. When significant effects were found, effect sizes and 95% confidence intervals were computed using Cohen’s d^42^ plus a correction factor to account for the cross-over nature of the trial^43^ and assuming a correlation of 0.8 between visits.^44^ Effect sizes for differences between formulations were reported and defined as the following: trivial (<0.2), small (0.2–0.49), medium (0.5–0.79) or large (≥0.8).^42^

### Role of funders

Funders of this research had no role in study design, data collection, data analyses, interpretation, or writing of the manuscript.

## Results

### Trial population and participant flow

Completer participants were 53 healthy adults (62% women, 38% men) with overweight or obesity, whose baseline characteristics are compiled in Table 2. Recruitment for the trial commenced in May 2021 and the LPLV was early October 2022. There were no differences in descriptive characteristics of participants between the intervention sites. Participants were predominantly white European. Biochemistry results were within the normal to moderate range.

**Table 2:** Sample Description for whole sample and by intervention site. Data are shown as mean (SD) and median [interquartile range].

	ALL	UNIVLEEDS	CRNH
Sex:			
Female (n)	33	20	13
Male (n)	20	9	11
Age (years)	45 [17]	46 [15]	39.5 [20]
Ethnicity:			
White European	41	23	18
Black African & Black other	5	2	3
Asian	3	3	0
Other ethnic groups	2	1	1
Unknown/prefer not to answer	2	0	2
Weight at baseline (kg)	81.7 (10.7)	80.7 (11.7)	83.0 (9.4)
Height (cm)	169.0 (8.3)	169.0 (8.2)	168.9 (8.6)
BMI (kg/m^2^)	28.6 (2.7)	28.2 (3.0)	29.1 (2.3)
EAT-26 score (0–78)	3.0 [4.0]	3.0 [4.0]	2.0 [6.0]
sFFQ score (0–12)	9.0 [2.0]	9.0 [2.0]	9.0 [3.0]
Waist circumference (cm)	96.6 (9.8)	94.9 (10.1)	98.6 (9.3)
Hip circumference (cm)	106.9 (6.5)	105.2 (5.7)	108.9 (6.8)
Waist-Hip Ratio (cm)	0.9 [0.1]	0.9 [0.2]	0.9 [0.1]
Fasting glucose (mg/dL)	94.4 (8.38)	94.28 (8.33)	94.59 (8.62)
Fasting insulin (μU/mL)	7.71 (3.92)	7.54 (3.61)	7.87 (4.27)
Fasting triglycerides (mg/dL)	112.87 (48.62)	114.53 (54.59)	111.21 (43.01)
Fasting total cholesterol (mg/dL)	190.61 (29.30)	187.72 (28.01)	193.50 (30.88)
Fasting HDL-cholesterol (mg/dL)	49.54 (10.20)	50.61 (12.27)	48.46 (7.74)
Fasting LDL-cholesterol (mg/dL)	103.72 (24.42)	100.76 (22.11)	106.67 (26.69)
Fasting AST (IU/L)	19.13 (8.50)	18.33 (4.29)	19.93 (11.31)
Fasting ALT (IU/L)	21.84 (18.97)	19.88 (7.11)	23.80 (26.03)
Fasting GGT (IU/L)	21.96 (13.15)	17.14 (7.86)	26.78 (15.60)
Triglyceride-Glucose (TyG) index	4.59 (0.22)	4.60 (0.24)	4.59 (0.20)
Fatty Liver (FL) index	48.65 (22.95)	44.70 (25.00)	52.60 (20.49)
HOMA-IR	1.69 (1.01)	1.55 (0.81)	1.83 (1.17)
Physical activity (IPAQ, Total MET-minutes/week)	4068 [4330]	3914 [3760]	4120 [4537]
Liking of control biscuit (Taste test, 100 mm VAS)	83 [27]	76 [38]	83.5 [19]

### Primary outcome—composite appetite score

**Fig. 3:**
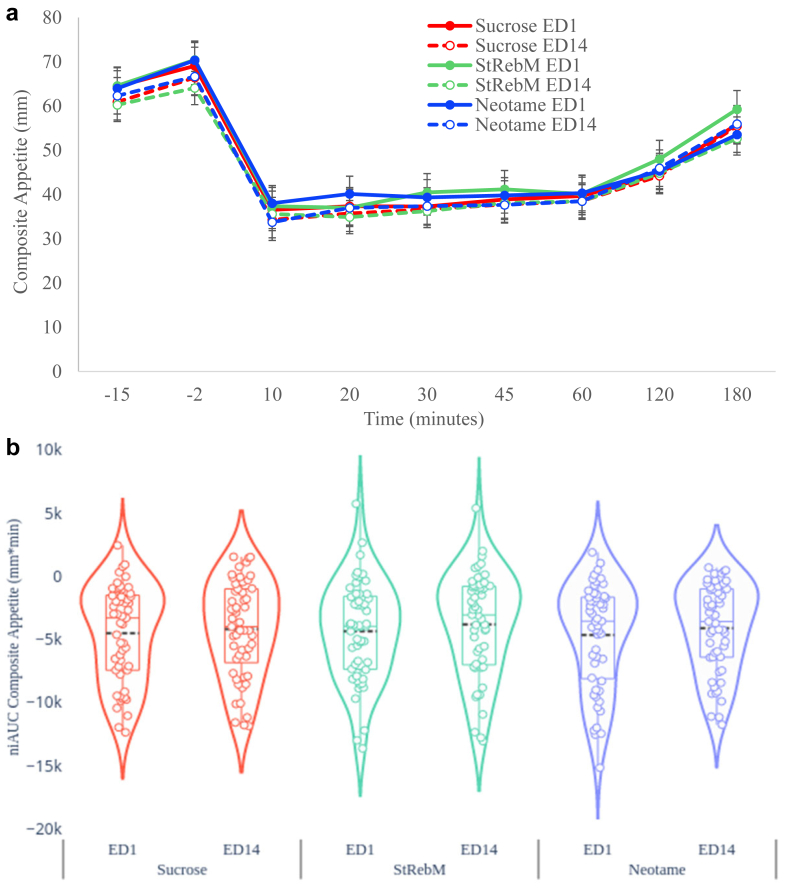
a: Composite appetite scores estimated marginal means according to S&SE formulation, exposure day (ED) and time using linear mixed models (n = 53). Error bars denote standardised error of the mean. b: Violin plots to show niAUC Composite appetite according to S&SE formulation and exposure day.

As shown in Fig. 3b and Supplementary Material S3, the linear mixed model revealed there was no overall effect of S&SE formulation (F (2, 104) = 0.54, p = 0.583) with similar niAUC composite appetite scores between sucrose, StRebM and Neotame. There was an overall effect of ED (F (1, 156) = 4.28, p = 0.040) with lower niAUC composite appetite scores on ED1 compared to ED14 (mean Δ −468.14 mm∗min, SEM 226.38; 95% CI (−915.31, −20.98)). Lower values indicate greater suppression of appetite. There was no formulation by ED interaction (F (2, 156) = 0.11, p = 0.895). Fig. 3a shows the profile of appetite change across the ED for illustrative purposes, while the analyses reported above are visualised in Fig. 3b as per the a priori statistical analysis plan.

### Other appetite sensations

#### Appetite for savoury or sweet

The linear mixed model revealed there was no effect of S&SE (F (2, 104) = 1.70, p = 0.188) with similar niAUC appetite for something savoury across formulations. There was no effect of ED (F (1, 156) = 0.22, p = 0.640) and no formulation by ED interaction (F (2, 156) = 0.45, p = 0.639). Similarly, there was no effect of formulation (F (2, 104) = 2.06, p = 0.133) on niAUC appetite for something sweet. There was an overall effect of ED (F (1, 156) = 7.82, p = 0.006) with lower appetite for sweet after ED1 compared to ED14 (mean Δ −843.03, SEM 301.39; 95% CI (−1438.36, −247.69)). There was no formulation by ED interaction (F (2, 156) = 1.27, p = 0.283). For data table see Supplementary Material S4.

#### Thirst, nausea and bloating

The linear mixed model revealed there was no effect of S&SE (F (2, 104) = 1.65, p = 197) or ED (F (1, 156) = 2.14, p = 0.146) on niAUC thirst values. There was a formulation by ED interaction on niAUC for thirst (F (2, 156) = 4.29, p = 0.015) with greater sensations of thirst after StRebM compared to sucrose on ED14 (mean Δ 1258.98 mm∗min, SEM 506.34; 95% CI (261.39, 2256.55)). There was also greater niAUC thirst values on ED14 compared to ED1 in the Neotame formulation (mean Δ 1217.84 mm∗min, SEM 471.08; 95% CI (287.32, 2148.36)).

The linear mixed model revealed there were no effects of formulation (F (2, 104) = 1.40, p = 0.250) or ED (F (1, 156) = 0.01 p = 0.938) on niAUC nausea values and no formulation by ED interaction (F (2, 156) = 1.08, p = 0.342).

While the linear mixed model revealed there were no effects of formulation (F (2, 104) = 2.13, p = 0.124) or ED (F (1, 156) = 1.30, p = 0.256) on niAUC for bloating, there was a formulation by ED interaction (F (2, 156) = 5.72, p = 0.004) with higher sensations of bloating reported in the 3 h after StRebM compared to sucrose on ED14 (mean Δ −1017.92 mm∗min, SEM 332.79; 95% CI (−1673.49, −362.34) and Neotame compared to sucrose on ED14 (mean Δ −960.06 mm∗min (units), SEM 332.79; 95% CI (−1615.63, −304.49). There were also lower niAUC bloating values on ED14 compared to ED1 with the sucrose formulation (mean Δ −1017.52 mm∗min (units), SEM 318.58; 95% CI (−1646.82, −388.223)). Data table can be found in Supplementary Material S5.

### Impact on subsequent food preferences

#### Liking and wanting for fatty or sweet foods

The linear mixed model revealed there was no effect of S&SE formulation (F (2, 104) = 2.96, p = 0.056) on pre-post intake change in explicit liking for high relative to low fat foods and no effect of ED (F (1, 156) = 0.14, p = 0.709). There was no formulation by ED interaction (F (2, 156) = 0.72, p = 0.490). Similarly, for pre-post intake change in explicit liking for sweet relative to savoury foods, there was no effect of formulation (F (2, 260) = 0.96, p = 0.383), ED (F (1, 260) = 0.17, p = 0.684) or formulation by ED interaction (F (2, 260) = 1.04, p = 0.356).

For pre-post intake change in implicit wanting for high fat relative to low fat foods, there was no effect of formulation (F (2, 260) = 1.89, p = 0.153), ED (F (1, 260) = 2.54, p = 0.112) or formulation by ED interaction (F (2, 260) = 0.49, p = 0.612). Similarly, there was no effect of formulation (F (2, 104) = 0.22, p = 0.800), ED (F (1, 156) = 0.64, p = 0.349) or formulation by ED interaction (F (2, 156) = 1.059, p = 0.349) on pre-post intake change in implicit wanting for sweet relative to savoury foods. See Supplementary Material S6 for data table and figures.

### Glycaemic and endocrine responses

#### Glucose and insulin

**Fig. 4:**
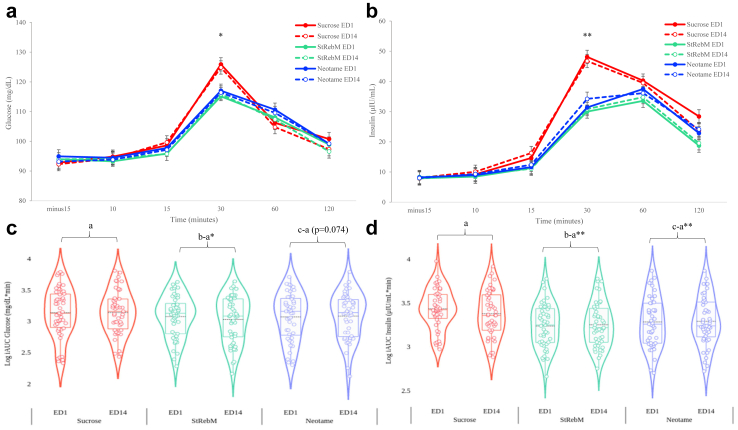
Glucose (a) and Insulin (b) levels estimated marginal means according to formulation, exposure day (ED) and timepoint analysed using linear mixed models (n = 47). Error bars denote standardised error of the mean. Violin plots to show Glucose (c) and Insulin (d) iAUC levels according to formulation and exposure day (ED). Biological samples were measured in duplicate samples. ∗p < 0.05, ∗∗p < 0.001.

As seen in Fig. 4, the linear mixed model showed an overall effect of S&SE formulation (F (2, 94) = 3.557, p = 0.032) with greater iAUC glucose values after the sucrose formulation compared to the StRebM (mean Δ 0.097 mg/dL∗min, SEM 0.037; 95% CI (0.023, 0.171); p < 0.05; d = −0.39). Differences between sucrose and Neotame formulations ran in the same direction but did not reach the statistical threshold (mean Δ 0.069 mg/dL∗min, SEM 0.038; 95% CI (−0.007, 0.145); p = 0.074; d = −0.25). There was no effect of ED (F (1, 140) = 0.038, p = 0.845) with similar iAUC glucose values on ED1 and ED14. There was no formulation by ED interaction (F (2, 139) = 0.449, p = 0.639).

There was an overall effect of formulation (F (2, 228) = 47.197, p < 0.001) on insulin response with greater iAUC insulin after the sucrose formulation compared to StRebM (mean Δ 0.169 μIU/mL∗min, SEM 0.018; 95% CI (0.133, 0.205); p < 0.001; d = −1.01) and Neotame (mean Δ 0.130 μIU/mL∗min, SEM 0.018; 95% CI (0.093, 0.166); p < 0.001; d = −0.71). There was no overall effect of ED (F (1, 228) = 0.149, p = 0.699) or formulation by ED interaction (F (2, 228) = 1.813, p = 0.165). Further details in Supplementary Material S7.

#### Ghrelin, GLP-1 and PP

**Fig. 5:**
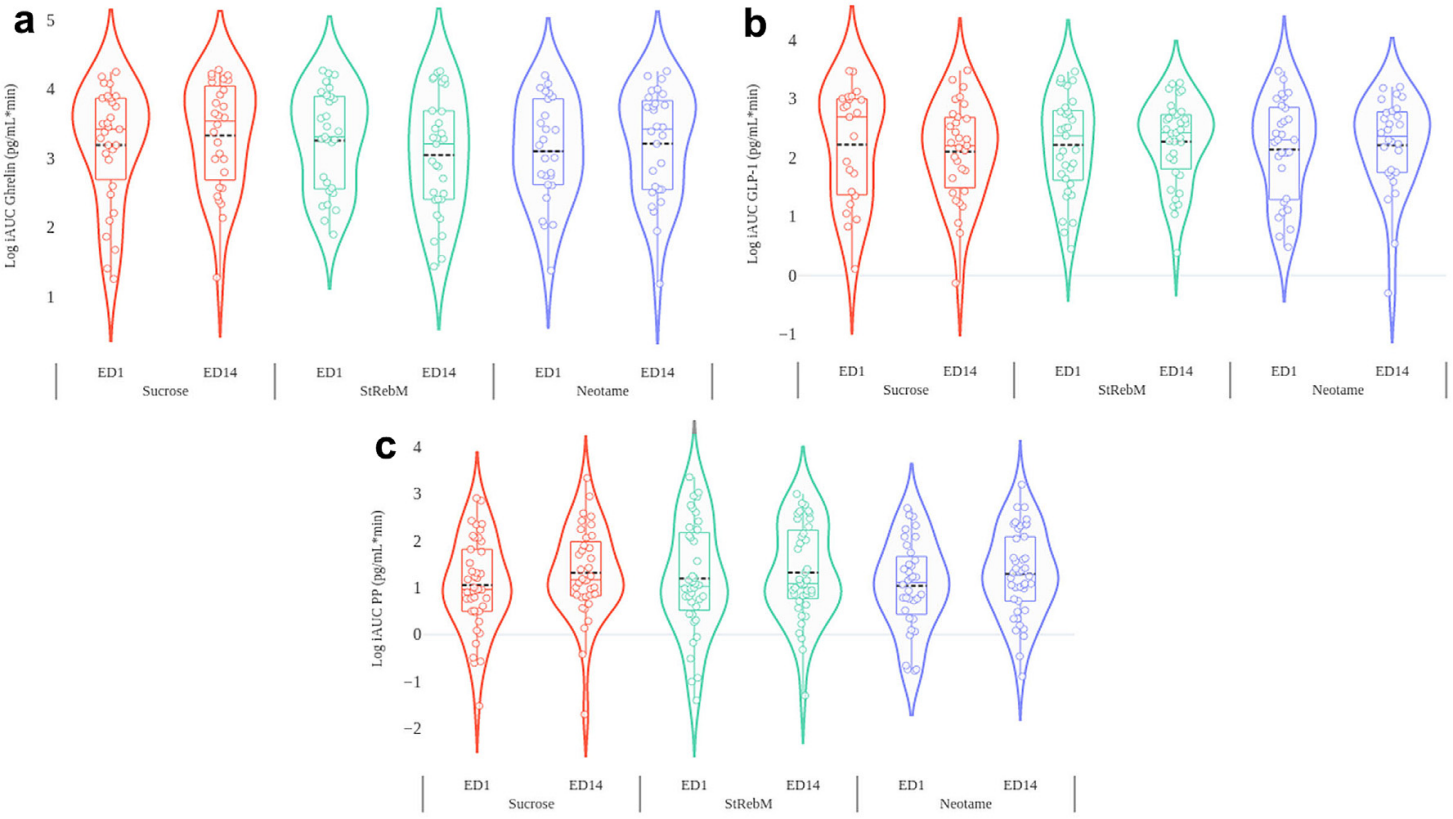
iAUC Ghrelin (a), GLP-1 (b) and PP (c) levels estimated marginal means according to formulation and exposure day analysed using linear mixed models (n = 40). Biological samples were measured in duplicate samples.

As shown in Fig. 5, the linear mixed model showed there was no effect of formulation (F (2, 78) = 0.436, p = 0.648), ED (F (1, 99) = 0.002, p = 0.963) or formulation by ED interaction (F (2, 198) = 0.688, p = 0.505) on the iAUC ghrelin response.

Similarly, there was no effect of formulation (F (2, 81) = 0.089, p = 0.915), ED (F (1, 88) = 0.087, p = 0.769) or formulation by ED interaction (F (2, 87) = 0.383, p = 0.683) on the iAUC GLP-1 response.

Lastly, there was no effect of formulation (F (2, 183) = 0.019, p = 0.981), but there was an overall effect of ED (F (1, 181) = 5.26, p = 0.023), with higher PP iAUC values on ED14 compared to ED1 (mean Δ 0.222 pg/mL∗min, SEM 0.097; 95% CI (0.031, 0.413)). There was no formulation by ED interaction (F (2, 182) = 0.185, p = 0.831) on the iAUC PP response. Full details in Supplementary Material S8.

### Gastrointestinal (GI) symptoms reported during the intervention periods

The frequencies of reported GI symptoms are shown in Table 3. Thirty-six of the 53 participants (67.9%) reported experiencing at least one GI symptom during the at-home intervention periods. Of the participants who reported GI symptoms, the most frequently reported symptoms were experienced in the Neotame formulation, followed by StRebM formulation and then sucrose, with the most frequently reported symptom being excess flatulence.

**Table 3:** Frequency of GI symptoms reported during the at-home intervention periods.

	Frequency of symptoms experienced			Frequency of symptoms thought to be related to study foods		
	Sucrose	StRebM	Neotame	Sucrose	StRebM	Neotame
Total	165	392	454	24	44	49
1. Abdominal pain/cramps not related to menstruation	3	8	11	1	3	0
2. Heartburn	2	0	5	0	0	0
3. Stomach acid/reflux	8	2	4	0	0	0
4. Nausea	5	3	6	0	0	0
5. Vomiting	4	3	2	0	0	0
6. Abdominal rumbling	5	29	32	0	1	0
7. Bloating	9	65	74	0	6	14
8. Belching	5	13	6	0	3	0
9. Excess gas/wind	24	100	124	12	15	24
10. Opened bowels less frequently than usual	12	1	16	0	0	11
11. Opened bowels more frequently than usual	6	32	33	0	0	0
12. Stool type	61	83	103	0	2	0
13. Urgent need for using the toilet	5	45	25	0	14	0
14. Stool retention sensation	16	8	13	11	0	0

Note—12. Stool type thought to be related to study food was identified twice as type 6 (mushy stool) on Bristol Stool Chart by one participant.

## Discussion

This study sought to evaluate the acute and two-week daily consumption effects of reformulated, no added sugar, solid food containing either Neotame or Stevia Rebaudioside M, compared to sucrose sugar-containing control on appetite and endocrine responses in adult men and women with overweight and obesity. The primary outcome, was the composite appetite score which showed there were no differences between the S&SE formulations on appetite sensations. The secondary findings on food preferences revealed no differences in pre-to post-intake changes in explicit liking or implicit wanting for high fat relative to low fat, or sweet relative to savoury foods, and no differences after acute vs. repeated exposure. Results showed insulin responses were lower after the StRebM and Neotame formulations compared to sucrose and glucose responses were lower after StRebM compared to sucrose. With blood glucose, the difference between sucrose and neotame was similar to sucrose vs. StRebM, but did not reach significance. There were no differences between formulations on ghrelin, GLP-1 or PP, nor an effect of acute vs. repeated exposure on ghrelin or GLP-1 60 min responses, indicating that S&SE did not decrease the early intestinal satiety response. There was however an increase in PP iAUC values measured up to 30 min post-ingestion after repeated exposure, derived mostly from increases in peak values after sucrose intake on day 14. Gastrointestinal symptoms (the most commonly reported symptom being flatulence), were most frequently reported in the Neotame formulation, followed by StRebM, with relatively few symptoms reported in the intervention period of the sucrose formulation.

The absence of differences between formulations on composite appetite scores revealed that the biscuits reformulated with StRebM or Neotame produced a similar acute reduction in appetite compared to biscuits formulated with sucrose. The sucrose biscuit contained ≈ 30 kcal more energy than the biscuits sweetened with StRebM or Neotame, but the additional calories provided from sucrose would not be expected to affect satiety levels over 180 min. There are a lack of studies investigating StRebM or Neotame, but we can consider studies of the effects of all stevia compounds and, cautiously, aspartame on appetite and endocrine variables. The recent systematic review and meta-analysis commissioned by the WHO on the health effects of non-sugar sweeteners^45^ included five RCTs evaluating the effects of Aspartame and Stevia/StRebA/Steviol glycosides on hunger, satiety, and appetite.^18^^,^^46^, ^47^, ^48^, ^49^ They reported no effect of S&SE on hunger, a weaker effect on satiety and increase in appetite/desire to eat. However, these analyses did not account for differences in energy content of the no S&SE comparison formulations (water or sucrose) and only one study, investigating the effects of aspartame on appetite-related outcomes, included aspartame-sweetened solid foods.^47^ The results from the current study indicate no effect of StRebM on appetite however, Ahmad et al. reported that incorporation of Stevia leaf powder in a cookie reduced feelings of hunger at 30 min, compared to a control cookie without Stevia.^50^ In other studies using beverages, Stevia led to lower subjective feelings of hunger and desire to eat immediately after consumption, and a lower VAS score for hunger before lunch compared to water^51^ and showed beneficial effects on appetite and energy intake when consumed prior to a meal.^52^ On the other hand, the results on the effect of Neotame on appetite from the current study are in accordance with those of previous work on aspartame, which showed no change in hunger when aspartame was added to energy-yielding foods, beverages, or meals.^46^^,^^47^^,^^53^ Differences in findings with the present study may be due to the primary outcome being niAUC for composite appetite score vs. time-point and VAS sensation specific effects in the previous literature. Recently, another study from the SWEET consortium examined acute effects of S&SE delivered in flavoured beverages and no differences were identified between the niAUC for appetite and satiety ratings for a StRebM with Mogroside V blend, vs. the sucrose control.^54^

Differences in niAUC appetite score were detected for repeated vs. acute exposure in the current study, with less suppression of appetite after two-weeks of consumption. This difference however did not differ between the 3 formulations. Findings across the secondary behavioural outcomes (nausea, bloating, thirst, appetite for savoury/sweet) align with the primary outcome of composite appetite score.

There is uncertainty whether S&SE impact the central taste and reward centres in the brain in the same manner as caloric sweeteners,^16^ and as to whether the reinforcing effects of a caloric load are derived from the interaction of nutrients with sensors in the gut lumen, or metabolic responses to specific nutrients^55^ including interactions with the gut microbiota.^56^^,^^57^ Food reward is often conceptualised as two sub-components—liking and wanting—meaning that sensory (e.g., sweet taste) and post-ingestive factors (e.g., energy content) can independently modulate dopamine levels in brain reward circuits.^58^ In the present study, there were no pre-to post-intake differences in either explicit liking or implicit wanting for high fat relative to low fat or sweet relative to savoury foods. A previous study, employing the same food reward methodology, reported a greater acute reduction in liking and wanting for high fat foods following consumption of a high carbohydrate compared to a high fat meal, otherwise matched for energy content, sensory properties, and appearance.^59^ A reduced preference for high fat foods after food intake would be consistent with an overall suppression of motivation to eat, but was not observed in the present study. This could be due to the relatively modest energy content of the biscuit formulations.

Both StRebM and Neotame reformulations resulted in a lowered insulin response compared to the sucrose control formulation. Similar patterns were seen for glucose, i.e., StRebM and Neotame resulted in lower iAUC glucose, but this did not reach significance for Neotame vs. sucrose. These findings support the findings of several studies on steviol glycosides (mostly StRebA)^20^^,^^52^^,^^60^ although the present study adds to these findings given that it used a solid food matrix and more equal calorie loads across the biscuit formulations than previous studies. No effect of formulation was shown for ghrelin or GLP-1 after acute or repeated exposure, but there was an effect of repeated exposure on PP levels, with higher levels found after 14 days. This is in agreement with studies on sucralose which have shown no effect on GLP-1,^61^^,^^62^ PYY,^61^, ^62^, ^63^ GIP,^63^ or acylated ghrelin.^64^ Similarly aspartame has been shown to have no effect on PYY or GLP-1.^62^

The finding that GI symptoms were more frequently reported in Neotame and StRebM formulations may be due to the presence of polyols in relatively higher amounts in the reformulated biscuits than the control. Polyols are classed as low digestible carbohydrates (LDCs) since they are incompletely absorbed in the small intestine, and are at least partially fermented by bacteria in the large intestine.^65^ As a result of this fermentation, LDCs can affect laxation and produce GI effects such as abdominal discomfort and flatulence.^66^ The observed reduction in sensations of bloating over the intervention period could be explained by the fact that some individuals may adapt to intake of LDCs over time, due to increased fermentation capacity of colonic bacteria.^66^

Strengths of the study include that it was highly controlled and used a standardised pre-published protocol with common training of procedures across intervention centres. Use of a crossover design allowed the control of intra-individual differences. A wide range of endpoints were considered, made feasible by the multidisciplinary approach to the study, the relatively large sample size and inclusion of relevant covariates compared to similar studies. Food form and structure affects digestion profile and postprandial responses^67^; for example, there are differences in the relative satiating power of liquid compared to solid energy preloads including altered appetite and hormonal responses as well as energy compensation.^68^, ^69^, ^70^ It follows therefore that results from studies on S&SE conducted in beverage matrices have limited applicability to solid foods and highlights the unique nature of the current study. Studying the effects of S&SE specifically in a cohort with overweight/obesity has been identified as a critical research need,^71^ which was achieved in this study.

A limitation of this study design is the necessary use of added polyols in the reformulated products. For the biscuits to be matched as much as possible for energy content as well as for taste and organoleptic properties, it was necessary to use ingredients that match the functionality of sucrose. Addition of ≈ 8% maltitol and ≈ 8% sorbitol, resulted in biscuits that were very well matched for sensory properties. Moreover, finding viable alternatives to polyol bulking agents in achieving sugar reduction in solid food matrices such as biscuits and cakes is considerably challenging,^11^ and currently not possible.

Overall, the results of this study suggest there are no acute or repeated consumption differences between Neotame, StRebM or sucrose on appetite or satiety-related endocrine responses when consumed in a solid food matrix. Nevertheless, glucose and insulin responses were blunted after acute and repeated consumption of S&SE reformulated biscuits, which may confer a benefit for blood glucose control, for example in individuals at risk of developing type 2 diabetes.

## Contributors

JAH, JCGH, and ARA are the SWEET project coordinators. JAM and GF are leader and co-leader for this clinical trial work package in SWEET; CS coordinated the S&SEs selection process which enable their inclusion into the biscuits. CR and ALB tested and produced the intervention biscuit products. GF, CG, KB, and J-AN led the intervention studies at Leeds and CRNH with support from BOH, DOC, EAR, SNC, CH, MW and LK. CG completed data analysis with KB accessing and verifying the data. HM performed the biochemical analyses. CG wrote the first draft of the paper. All authors provided revisions and have approved the final version of the manuscript. The wider consortium provided advice on product development and study design prior to starting the study.

## Data sharing statement

Pseudoanonymised data are stored in a secure datahub managed at University of Navarra as specified in the study protocol. Anonymised data will be archived in a publicly available repository from 2028 (e.g., UK Data Service). Data underlying this research are available from the corresponding author upon reasonable request and approval from the project coordinators.

## Declaration of interests

JCGH and JAH are in receipt of research funding from the American Beverage Association. ARA has received honoraria from Nestle, Unilever and the International Sweeteners Association. University of Liverpool has received income from International Food Information Council by CH. CH has received honoraria for work with Food Standards Agency Advisory Committee on Social Sciences. MW was previously contracted to research funded by AstraZeneca through funding paid to University of Liverpool. CS is a paid employee of Cargill, Inc. The University of Leeds has received income from consultancy for Mars Inc by JCGH. EAR has received honoraria for manuscript writing from Institute of Life Sciences (ILSI-Europe).

## Appendix ASupplementary data

Supplementary Materials S1–S8

Gibbons et al 2022

List of consortia members

## Footnotes

m The United States Food and Drink Administration approved Neotame for use as a food additive in 2002 and granted StRebM ‘Generally Recognised as Safe’ status in 2019; the European Food Safety Authority approved StRebM in 2019 and is currently reviewing Neotame. Neotame is currently under review by EFSA. Neotame was evaluated by the EU Joint Expert Committee on Food Additives in 2003 and assigned an ADI of 0–2 mg/kg body weight.
