# Supplementary material for: Acute and two-week effects of neotame, stevia rebaudioside M and sucrose-sweetened biscuits on postprandial appetite and endocrine response in adults with overweight/obesity—a randomised crossover trial from the SWEET consortium
Source: eBioMedicine. 2024 Mar 28;102:105005. doi: 10.1016/j.ebiom.2024.105005 (PMC11026940; doi:10.1016/j.ebiom.2024.105005)
Supplement: Supplementary Materials S1–S8 [file mmc1.docx]

**Table of Contents of Supplementary Material**

Supplementary Material 1: Image of study biscuits…………………………………...Page 2

Supplementary Material 2: Calculations used for Data Processing……………………Page 3

Supplementary Material 3: Data table for niAUC composite appetite………………...Page 4

Supplementary Material 4: Data table for niAUC appetite for savoury and sweet…….Page 5

Supplementary Material 5: Data table for niAUC for thirst, nausea and bloating……..Page 6

Supplementary Material 6: Data table and figures for food preferences……………….Page 7

Supplementary Material 7: Data table for iAUC glucose and insulin………………….Page 8

Supplementary Material 8: Data table for ghrelin, GLP-1 and PP……………………..Page 9

**Supplementary Material 1: Image of the study biscuits (control version)**


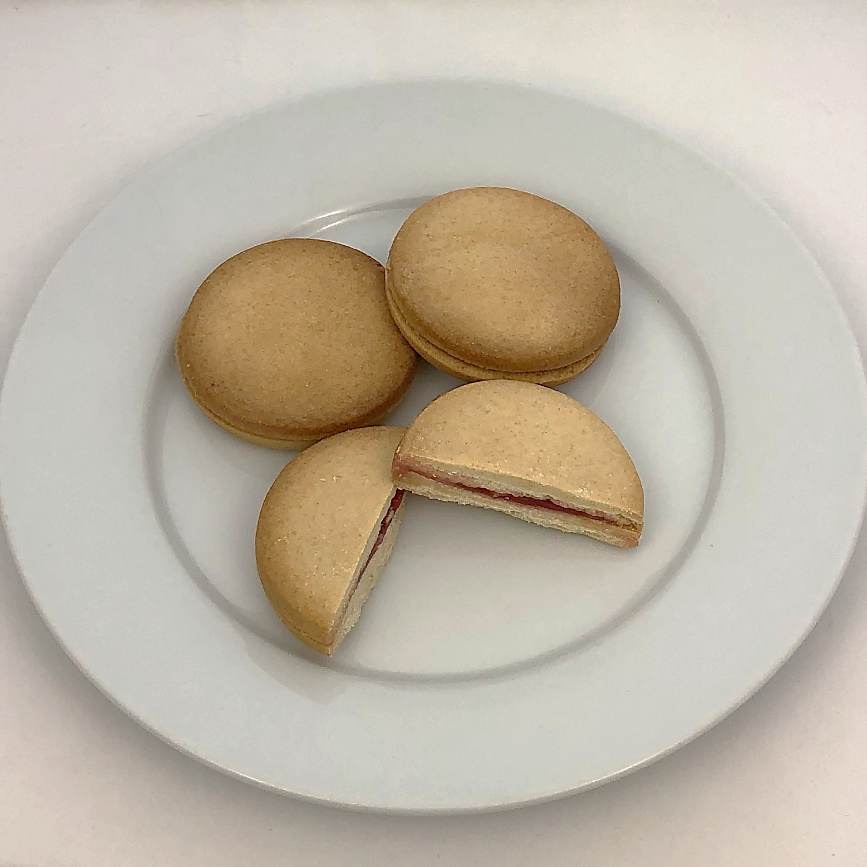


**Supplementary Material 2: Calculations used for Data Processing**

**Calculation of Composite Appetite Score^1^:**

Appetite score = [desire to eat + hunger + (100 − fullness) + prospective consumption]/4

All variables are collected using 100 point VAS.

**Calculation of Fatty Liver Index^2^:**

Some of the blood parameters will be used to calculate a Fatty Liver index (FL) using the formula of Bedogni et al ^1^,with measured values for BMI, fasting TG (mg/dL), fasting GGT (U/L) and waist circumference (cm), as follows:


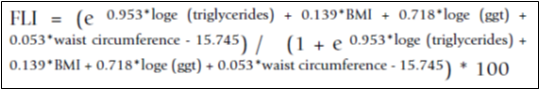


**Calculation of Triglyceride Glucose Index^3^:**

The formula of Simental-Mendía et al.^2^ will be measured with measured fasting TG (mg/dL) and fasting glucose (mg/dL), by dividing the Ln of the TG *glucose product by 2:

TyG index = Ln [(fasting triglycerides) (mg/dL) x fasting glucose (mg/dL)] / 2

**Calculation of HOMA^4^:**

Glucose (mg/dL) * Insulin (μIU/mL) /450

**References:**

1. Anderson, G.H., Catherine, N.L.A., Woodend, D.M., & Wolever, T.M.S. (2002) Inverse association between the effect of carbohydrates on blood glucose and subsequent short-term food intake in young men. *The American Journal of Clinical Nutrition, 76*(5) 1023-1030. <https://doi.org/10.1093/ajcn/76.5.1023>
2. Bedogni, G., Bellentani, S., Miglioli, L., Masutti, F., Passalacqua, M., Castiglione, A., & Tiribelli, C. (2006). The fatty liver index: A simple and accurate predictor of hepatic steatosis in the general population. *BMC Gastroenterology*, *6*(1), 33. <https://doi.org/10.1186/1471-230X-6-33>
3. Simental-Mendía, L. E., Rodríguez-Morán, M., & Guerrero-Romero, F. (2008). The product of fasting glucose and triglycerides as surrogate for identifying insulin resistance in apparently healthy subjects. *Metabolic Syndrome and Related Disorders*, *6*(4), 299–304. [https://doi.org/10.1089/met.2008.0034](https://doi.org/10.1089/met.2008.0034%E2%80%AF)
4. Matthews, D.R., Hosker J. P., Rudenski, A. S., Naylor, B. A., Treacher D. F.,  Turner, R. C. (1985) Homeostasis model assessment: insulin resistance and β-cell function from fasting plasma glucose and insulin concentrations in man. *Diabetologia* volume 28, 412–419

**Supplementary Material 3: Data table for niAUC composite appetite**

niAUC composite appetite scores estimated marginal means with 95% confidence intervals and effects of formulation, exposure day (ED) and formulation by exposure day interactions from unadjusted and adjusted linear mixed models.

|  |  |  | Sucrose | | StRebM | | Neotame | | LMM fixed effects | | | | |
| --- | --- | --- | --- | --- | --- | --- | --- | --- | --- | --- | --- | --- | --- |
|  |  |  | ED1 | ED14 | ED1 | ED14 | ED1 | ED14 |  | Unadjusted | | Adjusted^a^ | |
|  |  |  |  |  |  |  |  |  |  | *F* | *p* | *F* | *p* |
| niAUC composite appetite score (mm*min) | Mean | | -4485 | -4168 | -4328 | -3797 | -4636 | -4079 | Cond | 0.542 | 0.583 | - | - |
|  | SEM | | 536 | 536 | 536 | 536 | 536 | 536 | ED | 4.276 | 0.04 | - | - |
|  | 95% CI | lower | -5550 | -5233 | -5393 | -4862 | -5701 | -5144 | Cond*ED | 0.112 | 0.895 | - | - |
|  |  | upper | -3420 | -3103 | -3263 | -2732 | -3571 | -3014 |  |  |  |  |  |

^a^ No covariates were significant in the adjusted model

**Supplementary Material 4: Data table for niAUC appetite for savoury and sweet**

niAUC appetite for savoury and sweet estimated marginal means with 95% confidence intervals and effects of formulation, exposure day (ED) and formulation by exposure day interactions from unadjusted and adjusted linear mixed models.

|  |  |  | Sucrose | | StRebM | | Neotame | | LMM fixed effects | | | | |
| --- | --- | --- | --- | --- | --- | --- | --- | --- | --- | --- | --- | --- | --- |
|  |  |  | ED1 | ED14 | ED1 | ED14 | ED1 | ED14 |  | Unadjusted | | Adjusted^a^ | |
|  |  |  |  |  |  |  |  |  |  | *F* | *p* | *F* | *p* |
| niAUC appetite for savoury (mm*180min) | Mean | | -1582 | -1812 | -1275 | -910 | -1703 | -1457 | Cond | 1.699 | 0.188 | 1.699 | 0.188 |
|  | SEM | | 559 | 559 | 559 | 559 | 559 | 559 | ED | 0.22 | 0.64 | 0.22 | 0.64 |
|  | 95% CI | lower | -2691 | -2920 | -2384 | -2019 | -2812 | -2566 | Cond*ED | 0.448 | 0.639 | 0.448 | 0.639 |
|  |  | upper | -474 | -703 | -167 | 198 | -595 | -349 |  |  |  |  |  |
| niAUC appetite for sweet (mm*180min) | Mean | | -4841 | -4609 | -5393 | -3984 | -4469 | -3582 | Cond | 2.06 | 0.133 | - | - |
|  | SEM | | 612 | 614 | 612 | 612 | 612 | 612 | ED | 7.824 | 0.006 | - | - |
|  | 95% CI | lower | -6054 | -5826 | -6606 | -5197 | -5682 | -4795 | Cond*ED | 1.271 | 0.283 | - | - |
|  |  | upper | -3629 | -3391 | -4180 | -2772 | -3256 | -2369 |  |  |  |  |  |

^a^ BMI was a significant covariate in the adjusted model for appetite for savoury

**Supplementary Material 5: Data table for niAUC for thirst, nausea and bloating**

niAUC thirst, nausea and bloating scores estimated marginal means with 95% confidence intervals and effects of formulation, exposure day (ED) and formulation by exposure day interactions from unadjusted and adjusted linear mixed models.

|  |  |  | Sucrose | | StRebM | | Neotame | | LMM fixed effects | | | | |
| --- | --- | --- | --- | --- | --- | --- | --- | --- | --- | --- | --- | --- | --- |
|  |  |  | ED1 | ED14 | ED1 | ED14 | ED1 | ED14 |  | Unadjusted | | Adjusted^a^ | |
|  |  |  |  |  |  |  |  |  |  | *F* | *p* | *F* | *p* |
| niAUC thirst (mm*180min) | Mean | | -566.56 | -1247.55 | -1533.57 | -315.73 | -644.49 | 11.43 | Cond | 1.65 | 0.197 | - | - |
|  | SEM | | 479.02 | 479.02 | 479.02 | 479.02 | 479.02 | 479.02 | ED | 2.14 | 0.146 | - | - |
|  | 95% CI | lower | -1512.93 | -2193.92 | -2479.94 | -1262.10 | -1590.87 | -934.95 | Cond*ED | 4.29 | 0.015 | - | - |
|  |  | upper | 379.82 | -301.17 | -587.19 | 630.65 | 301.89 | 957.80 |  |  |  |  |  |
| niAUC nausea (mm*180min) | Mean | | -497.97 | -837.86 | -852.57 | -803.76 | -515.55 | -180.63 | Cond | 1.4 | 0.25 | - | - |
|  | SEM | | 297.67 | 297.67 | 297.67 | 297.67 | 297.67 | 297.67 | ED | 0.01 | 0.938 | - | - |
|  | 95% CI | lower | -1084.49 | -1424.37 | -1439.08 | -1390.27 | -1102.06 | -767.15 | Cond*ED | 1.08 | 0.342 | - | - |
|  |  | upper | 88.54 | -251.35 | -266.05 | -217.24 | 70.97 | 405.88 |  |  |  |  |  |
| niAUC bloating (mm*180min) | Mean | | 296.75 | -720.77 | -199.64 | 297.14 | 347.59 | 239.28 | Cond | 2.13 | 0.124 | - | - |
|  | SEM | | 269.06 | 269.06 | 269.06 | 269.06 | 269.06 | 269.06 | ED | 1.3 | 0.256 | - | - |
|  | 95% CI | lower | -233.29 | -1250.81 | -729.68 | -232.89 | -182.44 | -290.75 | Cond*ED | 5.72 | 0.004 | - | - |
|  |  | upper | 826.78 | -190.74 | 330.39 | 827.18 | 877.63 | 769.32 |  |  |  |  |  |

^a^ No covariates were significant in the adjusted model

**Supplementary Material 6: Data table and figures for food preferences**

Pre-post intake change in explicit liking fat bias, explicit liking sweet bias, implicit wanting fat bias and implicit wanting sweet bias scores (mm) estimated marginal means with 95% confidence intervals and effects of formulation, exposure day (ED) and formulation by exposure day interactions from unadjusted and adjusted linear mixed models.

|  |  |  | Sucrose | | StRebM | | Neotame | | LMM fixed effects | | | | |
| --- | --- | --- | --- | --- | --- | --- | --- | --- | --- | --- | --- | --- | --- |
|  |  |  | ED1 | ED14 | ED1 | ED14 | ED1 | ED14 |  | Unadjusted | | Adjusted^a^ | |
|  |  |  |  |  |  |  |  |  |  | F | p | F | p |
| EL fat bias | Mean | | -4 | -2 | -3 | -3 | -5 | -6 | Cond | 2.96 | .056 | 2.96 | .056 |
|  | SEM | | 1 | 1 | 1 | 1 | 1 | 1 | ED | 0.14 | .709 | 0.14 | .709 |
|  | 95% CI | lower | -7 | -5 | -5 | -6 | -8 | -9 | Cond*ED | 0.72 | .490 | 0.72 | .490 |
|  |  | upper | -1 | 1 | 0 | 0 | -3 | -3 |  |  |  |  |  |
| EL sweet bias | Mean | | -10 | -11 | -13 | -9 | -13 | -14 | Cond | 0.96 | .383 | - | - |
|  | SEM | | 3 | 3 | 3 | 3 | 3 | 3 | ED | 0.17 | .684 | - | - |
|  | 95% CI | lower | -16 | -17 | -19 | -15 | -19 | -20 | Cond*ED | 1.04 | .356 | - | - |
|  |  | upper | -3 | -5 | -7 | -2 | -6 | -7 |  |  |  |  |  |
| IW fat bias | Mean | | -9 | -8 | -8 | -2 | -11 | -8 | Cond | 1.89 | .153 | - | - |
|  | SEM | | 3 | 3 | 3 | 3 | 3 | 3 | ED | 2.55 | .112 | - | - |
|  | 95% CI | lower | -14 | -13 | -13 | -8 | -16 | -14 | Cond*ED | 0.49 | .612 | - | - |
|  |  | upper | -4 | -2 | -3 | 3 | -5 | -3 |  |  |  |  |  |
| IW sweet bias | Mean | | -17 | -20 | -22 | -15 | -18 | -15 | Cond | 0.22 | .800 | - | - |
|  | SEM | | 5 | 5 | 5 | 5 | 5 | 5 | ED | 0.64 | .426 | - | - |
|  | 95% CI | lower | -27 | -30 | -32 | -25 | -28 | -25 | Cond*ED | 1.06 | .349 | - | - |
|  |  | upper | -8 | -11 | -12 | -5 | -8 | -6 |  |  |  |  |  |

EL, explicit liking; IW, implicit wanting. ^a^Explicit liking fat bias: Age was a significant covariate in the adjusted model. Explicit liking sweet bias: No covariates were significant in the adjusted model. Implicit wanting fat bias: No covariates were significant in the adjusted model. Implicit wanting sweet bias: No covariates were significant in the adjusted model.

**Supplementary Material 7: Data table for iAUC Glucose and Insulin**

iAUC Glucose and Insulin estimated marginal means with 95% confidence intervals and effects of formulation, exposure day (ED) and formulation by exposure day interactions from unadjusted and adjusted linear mixed models.

|  |  |  | Sucrose | | StRebM | | Neotame | | LMM fixed effects | | | | |
| --- | --- | --- | --- | --- | --- | --- | --- | --- | --- | --- | --- | --- | --- |
|  |  |  | ED1 | ED14 | ED1 | ED14 | ED1 | ED14 |  | Unadjusted | | Adjusted^a^ | |
|  |  |  |  |  |  |  |  |  |  | *F* | *p* | *F* | *p* |
| iAUC Glucose (mg/dL*min) | Mean | | 3.13 | 3.14 | 3.06 | 3.02 | 3.06 | 3.08 | Cond | 3.56 | 0.032 | 3.59 | 0.031 |
|  | SEM | | 0.05 | 0.05 | 0.05 | 0.05 | 0.05 | 0.05 | ED | 0.04 | 0.845 | 0.04 | 0.836 |
|  | 95% CI | lower | 3.03 | 3.04 | 2.96 | 2.92 | 2.96 | 2.97 | Cond*ED | 0.45 | 0.639 | 0.45 | 0.638 |
|  |  | upper | 3.23 | 3.25 | 3.17 | 3.12 | 3.17 | 3.18 |  |  |  |  |  |
| iAUC Insulin (μIU/mL*min) | Mean | | 3.44 | 3.39 | 3.24 | 3.25 | 3.28 | 3.29 | Cond | 47.20 | <0.001 | 47.54 | <0.001 |
|  | SEM | | 0.04 | 0.04 | 0.04 | 0.04 | 0.04 | 0.04 | ED | 0.15 | 0.699 | 0.16 | 0.687 |
|  | 95% CI | lower | 3.36 | 3.32 | 3.16 | 3.18 | 3.20 | 3.22 | Cond*ED | 1.81 | 0.165 | 1.86 | 0.159 |
|  |  | upper | 3.51 | 3.46 | 3.31 | 3.32 | 3.35 | 3.36 |  |  |  |  |  |

^a^ BMI was a significant covariate in the adjusted models for iAUC Glucose and Insulin

**Supplementary Material 8: Data table for Ghrelin, GLP-1 and PP**

iAUC Ghrelin, GLP-1 and PP estimated marginal means with 95% confidence intervals and effects of formulation, exposure day (ED) and formulation by exposure day interactions from unadjusted and adjusted linear mixed models.

|  |  |  | Sucrose | | StRebM | | Neotame | | LMM fixed effects | | | | |
| --- | --- | --- | --- | --- | --- | --- | --- | --- | --- | --- | --- | --- | --- |
|  |  |  | ED1 | ED14 | ED1 | ED14 | ED1 | ED14 |  | Unadjusted | | Adjusted^a^ | |
|  |  |  |  |  |  |  |  |  |  | *F* | *p* | *F* | *p* |
| iAUC Ghrelin (pg/mL*min) | Mean | | 3.20 | 3.33 | 3.23 | 3.06 | 3.11 | 3.17 | Cond | 0.44 | 0.648 | - | - |
|  | SEM | | 0.14 | 0.15 | 0.15 | 0.14 | 0.15 | 0.15 | ED | 0.00 | 0.963 | - | - |
|  | 95% CI | lower | 2.93 | 3.04 | 2.94 | 2.78 | 2.80 | 2.87 | Cond*ED | 0.69 | 0.505 | - | - |
|  |  | upper | 3.48 | 3.62 | 3.51 | 3.34 | 3.41 | 3.46 |  |  |  |  |  |
| iAUC GLP-1 (pg/mL*min) | Mean | | 2.28 | 2.13 | 2.24 | 2.24 | 2.15 | 2.21 | Cond | 0.09 | 0.915 | - | - |
|  | SEM | | 0.16 | 0.14 | 0.14 | 0.14 | 0.15 | 0.15 | ED | 0.09 | 0.769 | - | - |
|  | 95% CI | lower | 1.96 | 1.85 | 1.97 | 1.97 | 1.86 | 1.92 | Cond*ED | 0.38 | 0.683 | - | - |
|  |  | upper | 2.60 | 2.42 | 2.52 | 2.51 | 2.44 | 2.51 |  |  |  |  |  |
| iAUC PP (pg/mL*min) | Mean | | 1.13 | 1.41 | 1.20 | 1.34 | 1.13 | 1.37 | Cond | 0.02 | 0.981 | - | - |
|  | SEM | | 0.15 | 0.16 | 0.15 | 0.15 | 0.16 | 0.15 | ED | 5.26 | 0.023 | - | - |
|  | 95% CI | lower | 0.82 | 1.09 | 0.90 | 1.04 | 0.81 | 1.07 | Cond*ED | 0.19 | 0.831 | - | - |
|  |  | upper | 1.43 | 1.72 | 1.51 | 1.65 | 1.45 | 1.67 |  |  |  |  |  |

^a^ No covariates were significant in the adjusted models
