# Supplementary material for: Acute and two-week effects of neotame, stevia rebaudioside M and sucrose-sweetened biscuits on postprandial appetite and endocrine response in adults with overweight/obesity—a randomised crossover trial from the SWEET consortium
Source: eBioMedicine. 2024 Mar 28;102:105005. doi: 10.1016/j.ebiom.2024.105005 (PMC11026940; doi:10.1016/j.ebiom.2024.105005)
Supplement: Gibbons et al 2022 [file mmc2.pdf]

BMJ Open

Acute and repeated impact of sweeteners and sweetness enhancers in solid and semi-solid foods on appetite: protocol for a multicentre, cross-over, RCT in people with overweight/obesity – the SWEET Project

Catherine Gibbons [1], Beverley O'Hara [1], Dominic O'Connor,[1] Charlotte Hardman,[2] Moon Wilton,[2] Joanne A Harrold,[2] Eva Almiron-Roig,[3,4] Santiago Navas-Carretero [3,4], Charo E Hodgkins [5], Julie Anne Nazare,[6] Maud Alligier,[6,7] Jose Alfredo Martínez,[3,8] Corey Scott,[9] Louise Kjølbæk [10], Mie Normand,[10] Cécile Rannou,[11] Ellen E Blaak [12], Edith Feskens,[13] Hariklia Moshoyiannis,[14] Anne Raben [10,15], Jason C G Halford,[1] Kristine Beaulieu [1], Graham Finlayson [1]

To cite: Gibbons C, O'Hara B, O'Connor D, *et al*. Acute and repeated impact of sweeteners and sweetness enhancers in solid and semi-solid foods on appetite: protocol for a multicentre, cross-over, RCT in people with overweight/obesity – the SWEET Project. *BMJ Open* 2022;**12**:e063903. doi:10.1136/bmjopen-2022-063903

► Prepublication history and additional supplemental material for this paper are available online. To view these files, please visit the journal online (http://dx.doi.org/10.1136/bmjopen-2022-063903).

CG and BO contributed equally.

Received 21 April 2022
Accepted 12 October 2022

Check for updates

© Author(s) (or their employer(s)) 2022. Re-use permitted under CC BY-NC. No commercial re-use. See rights and permissions. Published by BMJ.

For numbered affiliations see end of article.

Correspondence to
Dr Catherine Gibbons;
c.gibbons@leeds.ac.uk

ABSTRACT

Introduction Intake of free sugars in European countries is high and attempts to reduce sugar intake have been mostly ineffective. Non-nutritive sweeteners and sweetness enhancers (S&SEs) can maintain sweet taste in the absence of energy, but little is known about the impact of acute and repeated consumption of S&SE in foods on appetite. This study aims to evaluate the effect of acute and repeated consumption of two individual S&SEs and two S&SE blends in semisolid and solid foods on appetite and related behavioural, metabolic and health outcomes.

Methods and analysis A work package of the SWEET Project; this study consists of five double-blind randomised cross-over trials which will be carried out at five sites across four European countries, aiming to have n=213. Five food matrices will be tested across three formulations (sucrose-sweetened control vs two reformulated products with S&SE blends and no added sugar). Participants (body mass index 25–35 kg/m^2; aged 18–60 years) will consume each formulation for 14 days. The primary endpoint is composite appetite score (hunger, inverse of fullness, desire to eat and prospective food consumption) over a 3-hour postprandial incremental area under the curve during clinical investigation days on days 1 and 14.

Ethics and dissemination The trial has been approved by national ethical committees and will be conducted in accordance with the Declaration of Helsinki. Results will be published in international peer-reviewed open-access scientific journals. Research data from the trial will be deposited in an open-access online research data archive.

Trial registration number NCT04633681.

STRENGTHS AND LIMITATIONS OF THIS STUDY

- ⇒ The trial is the first of its kind to investigate the effects of acute and repeated exposure to two individual sweeteners and sweetness enhancers (S&SEs) and two S&SE blends in five different sweet food products across a variety of matrices including bakery (cakes and biscuits), dairy (yoghurt), confectionery (chocolate) and breakfast cereal.
- ⇒ This trial includes a large range of outcomes across behaviour, physiology and health from persons living in Northern, Central and Southern Europe.
- ⇒ The COVID-19 pandemic resulted in changes to the design of the studies in the trial. Originally, all products were to be tested across two sites, but the reduced time frame means this is not possible for some products.
- ⇒ Due to COVID-19 disruptions, the number of participants in two of the five studies will be reduced. Blood samples will not be taken in one of these smaller studies. Outcomes will be reported descriptively in these two studies where appropriate.

BACKGROUND AND RATIONALE

The global increase in the prevalence of obesity and its associated diseases is driven by a range of internal factors, involving genetic, behavioural and metabolic determinants along with permissive external factors from the physical, social and public (nutritional) policy environment.[1] One of the main behavioural drivers involves a diet too rich in energy intake relative to energy expenditure.

BMJ Open: first published as 10.1136/bmjopen-2022-063903 on 23 December 2022. Downloaded from http://bmjopen.bmj.com/ on January 5, 2023 by guest. Protected by copyright.

Free sugar intake (derived from sugar added to foods and beverages by the manufacturer or consumer) is one nutritional component that has gained focus because of its low nutritional value (lack of vitamins, minerals or fibre) and its potential to add to overall energy consumed, facilitating weight gain,[2] and potential altered appetite and endocrine responses to carbohydrates (sugars) relative to other macronutrients.[3]

Simply restricting free sugars from the diet without substitution may reduce diet palatability or contribute to changes in sweet craving,[4] particularly in women,[5] resulting in poor acceptance. The replacement of free sugars with non-nutritive sweeteners and sweetness enhancers (S&SEs) in food products is one method to reduce sugar intake while maintaining acceptance and palatability of the diet. S&SEs have increasingly been employed over recent years to reduce the energy and sugar content of foods; however, their impact on appetite-related and health-related outcomes is somewhat unclear.[6]

The effect of S&SEs on appetite is difficult to summarise due to the types of studies, comparisons and S&SEs being used. One of the reasons for the current partial understanding of the appetitive and metabolic effects of S&SEs in humans is that different S&SEs are commonly assumed to have similar behavioural effects.[6–8] Only recently, one 12-week investigation of four distinct S&SEs reported directionally dissimilar effects of saccharin compared with sucralose on body weight.[9] A recent review comparing different S&SEs suggests that some have the potential to enhance appetite, but these effects do not follow through to subsequent energy intake.[6] A recent meta-analysis detailed the impact of no-energy or low-energy sweetened preloads compared with conventionally sweetened preloads on ad libitum energy intake. They concluded that similar effects on energy intake were seen due to only partial compensation being evident (although total energy intake was lower in the no-energy or low-energy sweetener).[10] Furthermore, recent studies have highlighted that S&SEs may reduce sweet food cravings and therefore reduce sugar intake[11] appetite and energy intake.[12] Overall, there is currently insufficient evidence to make a clear conclusion about the effect of S&SEs on appetite and energy intake. Indeed, the 11 S&SEs that are currently approved for use in the European Union (EU) are chemically heterogeneous and absorbed, metabolised and excreted differently.[13] Furthermore, most investigations of the relationship between S&SE intake and health outcomes have used beverages as the vehicle[14]; these have recently been reviewed.[15] Since the amount of S&SEs in the food supply is increasing in response to consumer demand[16] and policy (eg, 'sugar taxes'[17 18]; EU initiatives[19 20]), there is a pressing need to examine the appetite-related behavioural and metabolic consequences of consuming S&SEs particularly in semi-solid and solid food matrices. Furthermore, it should be acknowledged that differences between acute and longer-term effects of S&SEs may not be the same[21] and this needs further investigation.

Aims and objectives

The main objective of this study is to evaluate the acute (1-day) and repeated (14-day) effects of two individual S&SEs and two S&SE blends in reformulated reduced or no added sugar food products (using two modulations of S&SEs per matrix) on appetite and related behavioural, metabolic and health outcomes in adult men and women with overweight or obesity.

The hypotheses are:

H_1: consumption of no added/reduced sugar products reformulated with S&SEs will result in an altered incremental area under the curve (iAUC) appetite score, compared with the sucrose-sweetened control product after repeated comparisons with acute consumption.

H_2: there will be differences between the no added/reduced sugar and sucrose-sweetened formulations on behavioural (eg, food reward and preferences, food cravings, self-reported energy intake), metabolic (satiety peptides, glycaemic and lipaemic response) and health-related (liver function and gastrointestinal (GI) side effects) outcomes.

Trial design

This study is part of the wider SWEET Project (https://sweetproject.eu/) funded by the European Commission Horizon 2020 programme. It is a multicentre double-blind, randomised cross-over trial conducted across five intervention sites in four countries, with three product formulations (sucrose-sweetened control vs two individual S&SEs or S&SE blends) over five intervention product types (cake, biscuits, yoghurt, chocolate and breakfast cereal matrices) aiming for a total of 213 completers. The protocol is reported as per the Standard Protocol Items: Recommendations for Interventional Trials reporting guidelines.[22] While this study addresses the short-term impact of specific S&SEs versus added sucrose on appetite, another work package in the SWEET Project will examine the long-term (1-year) impact of a weight loss and weight maintenance intervention with or without S&SE as part of a healthy diet.[23]

Sample size determination

The following calculations apply to the studies involving biscuit, yoghurt and chocolate matrices:

Primary outcome: power calculations showed that to detect a minimum difference of 8 mm in appetite ratings on a 100 mm Visual Analogue Scale (VAS) with 80% power, alpha 0.05 and based on a published within-subject SD of 14.4 mm in VAS measures,[24] an overall sample of 53 completers (both sexes, same body mass index (BMI) group, across all centres) would be needed[25] (p.30) per matrix. We expect this sample size will be sufficient to detect iAUC differences in the appetite response relative to control of around 8%–10%, considered to be of practical relevance.[26]

Secondary outcomes: due to the number of secondary outcomes in this study, it was not feasible to conduct

BMJ Open: first published as 10.1136/bmjopen-2022-063903 on 23 December 2022. Downloaded from http://bmjopen.bmj.com/ on January 5, 2023 by guest. Protected by copyright.

power calculations for all outcomes. However, we consulted published studies (eg, Yeomans *et al*[27]) which used a similar design and demonstrated effects of small nutritional manipulations on various gut peptides. In these studies, sample sizes ranged from 12 to 23 participants, giving us confidence that a sample of 53 participants per matrix should be sufficient to detect differences with clinical significance.

Due to the impact of the COVID-19 pandemic on the trial (detailed later), the cake and breakfast cereal studies were scaled down according to reduced feasibility at each intervention centre to n=24 (cake) and n=30 (breakfast cereal), and no blood samples will be collected in the cake study. The primary outcome will be reported descriptively in these two studies where appropriate and reflected in the study registration and protocol.

Study setting

This trial is conducted across five intervention sites in four countries across three regions of Europe, with each site testing a different product, while following the same protocol. Western Europe: Leeds (University of Leeds, UK) will test biscuits; Liverpool (University of Liverpool, UK) will test chocolate; Lyon (Centre de Recherche en Nutrition Humaine Rhône Alpes, France) will test biscuits and cakes; Northern Europe: Copenhagen (University of Copenhagen, Denmark) will test cereal; Southern Europe: Pamplona (University of Navarra, Spain) will test yoghurt. University of Leeds and University of Navarra are the leaders of this work package, while University of Liverpool is the coordinating centre of the SWEET Project in its entirety.

Patient and public involvement

During the study, research staff discuss with participants about their experiences of the clinical investigation days (CIDs), examinations, participant information, written materials, etc with the aim to understand and improve participants' experiences in current and future studies of this nature. This is also captured in an end of study survey.

Eligibility criteria

Male and female adults aged 18–60 years, with a BMI 25–35 kg/m^2, are eligible. Participants are required to regularly consume sugar-containing foods and willing to consume sugar and sweetened food products. During screening, they must have an Eating Attitudes Test (EAT-26)[28] score <20 and a short sweet food frequency questionnaire score ≥3 of 11, in addition to rating the control product as ≥40% on a 100-point liking VAS during the taste test and be willing to consume the product during the duration of the trial. All exclusion criteria are listed in online supplemental material 1.

Intervention

Each trial will begin with an initial exposure to one of the three assigned product formulations under controlled laboratory conditions (CIDs 1, 3, 5—exposure day 1), followed by repeated daily consumption of the same product at home for 12 (±2) days and a final exposure in the laboratory on day 14 (±2 days) under identical conditions as the first exposure (CIDs 2, 4, 6—exposure day 14), resulting in all participants completing the three product formulations in a Latin square design (see figure 1). CIDs 2 and 4 will be followed by a washout period of 14–21 days between formulations. During the at-home periods, participants will consume a portion of the product at a time and place they choose using a substitution strategy for similar energy/sweetness foods in their habitual diet. Foods habitually consumed of an equivalent energy density/sweetness are identified using participants' answers to a food frequency questionnaire and an energy equivalent guide, with a decision-making tree developed to identify the most suitable foods to substitute for each intervention product. This strategy is supported by advice and agreement from the research officer/dietitian. Compliance will be monitored by an intervention booklet completed daily and by return of empty food packaging. All food products are provided in the same blinded container/wrapping. The study duration for each participant will be a minimum of 70 days (plus 7–14 days' allowance for extended washout to aid scheduling of CIDs).

Recruitment and screening

Participants will be recruited via a variety of routes, for example, study databases, webpages, social media, posters and flyers. Potential participants will be prescreened using an online or telephonic prescreening questionnaire in accordance with the inclusion and exclusion criteria. Candidates passing prescreening will be invited to attend an information session, either online or in person, where they will be given detailed information about the study and invited to participate in a question and answer session. Candidates who wish to participate in the study will provide written informed consent and sign a general data protection regulation (GDPR) form before being fully screened. The screening session will be performed in person or online, and will consist of anthropometric measurements (height, weight, waist and hip circumference; all confirmed in person at CID1 for participants being screened online); eligibility questionnaires (EAT-26[28] and short sweet food frequency questionnaire); baseline questionnaires (a sociodemographic questionnaire, a questionnaire to assess habitual sweet food consumption, including regular and S&SE sweet foods (SWITCH sweet food frequency questionnaire),[29] a questionnaire to assess habitual physical activity (International Physical Activity Questionnaire[30] and a consumer perspective questionnaire); an eligibility taste test of the control intervention product where participants rated the pleasantness of the product on a 100 mm VAS after taking one bite and chewing for 5 s (a score of >40 mm was required for inclusion into the study). Candidates who pass the screening session will be enrolled into the study.

BMJ Open: first published as 10.1136/bmjopen-2022-063903 on 23 December 2022. Downloaded from http://bmjopen.bmj.com/ on January 5, 2023 by guest. Protected by copyright.

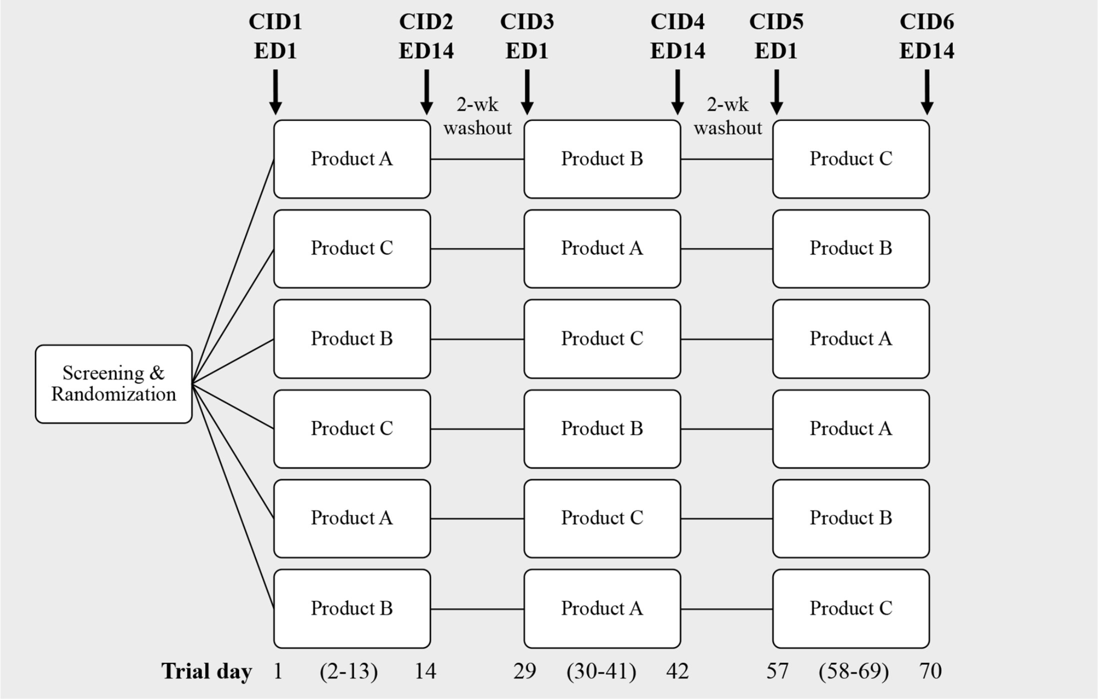

Figure 1 Latin square design and duration for cross-over trials. Each trial will include two no added/reduced sugar reformulated products and one sucrose-sweetened control (double-blind) per food matrix. Participants will be randomised to one of six treatment orders. For example, a participant randomised to order one will consume product A in the laboratory on clinical investigation day (CID) 1/exposure day (ED) 1 and then every day at home until CID2/ED14 when it is consumed in the laboratory again. After a 2-week washout, the participant returns to the laboratory and repeats the study block with product B, followed by another 2-week washout, followed by the final study block with product C.

Randomisation and blinding

A Latin square design (six treatment orders) will be used to randomly allocate product sequence into blocks of six, as shown in figure 1. The person responsible for generating the sequences for all sites will not have any study-related tasks, for example, inclusion or examination of participants. Each sequence will be stratified by sex (female/male) and age group (18–45 years/46–60 years). When feasible, a female/male ratio of minimum 60/40 was also considered to reflect the target population characteristics.

Blinding of the intervention products (reformulated and control products) will be done by the manufacturers. As such, blinding of the research and central laboratory staff will take place allowing for a double-blind intervention. Moreover, the statistical analyses of the main outcome variable will be done without breaking the intervention product-assignment code before the analyses are finalised.

Clinical investigation days

Prior to each CID, participants will be asked to consume a similar evening meal at the same time, before fasting for a minimum of 12 hours and a maximum of 15 hours. High-intensity physical activity, alcohol and coffee will not be allowed for 12 hours before arriving to the laboratory. Two glasses, approximately 500 mL, of non-carbonated water will be allowed during the fasting period. Participants will provide a spot urine sample collected maximum of 24 hours before each CID and will be analysed for the presence of specific S&SEs.

The CID procedures are outlined in figure 2. CID start times will be scheduled in the morning between 08:00 and 10:30 and participants will start all 6 CIDs at the same time. Participants will complete a protocol compliance questionnaire to verify the above requirements regarding diet, physical activity, etc. If compliance has been breached, staff will reschedule the CID (within the maximum 14 days allowed, otherwise a protocol deviation will be recorded). If compliance has been achieved, participants will then fill in the Control of Eating Questionnaire (CoEQ)[31] to assess cravings over the last 7 days, followed by a body weight measurement. Participants will consume 200 mL of water before having an intravenous cannula inserted into an antecubital vein by qualified personnel. A baseline fasting blood sample will be taken 15 min after insertion of the cannula. Once the fasting sample has been taken, participants will complete fasting subjective appetite ratings for hunger, fullness, thirst, desire to eat, prospective intake, nausea, bloating, appetite for something savoury and for something sweet on a validated 100-point VAS accessed via a personal computer or a tablet.[32 33] These measures will be completed on an electronic Questionnaire Delivery Platform (QDP), using separate screens for each VAS. Next, food reward will be measured using a culturally adapted version of the Leeds Food Preference Questionnaire (LFPQ)[34] on a computer

BMJ Open: first published as 10.1136/bmjopen-2022-063903 on 23 December 2022. Downloaded from http://bmjopen.bmj.com/ on January 5, 2023 by guest. Protected by copyright.

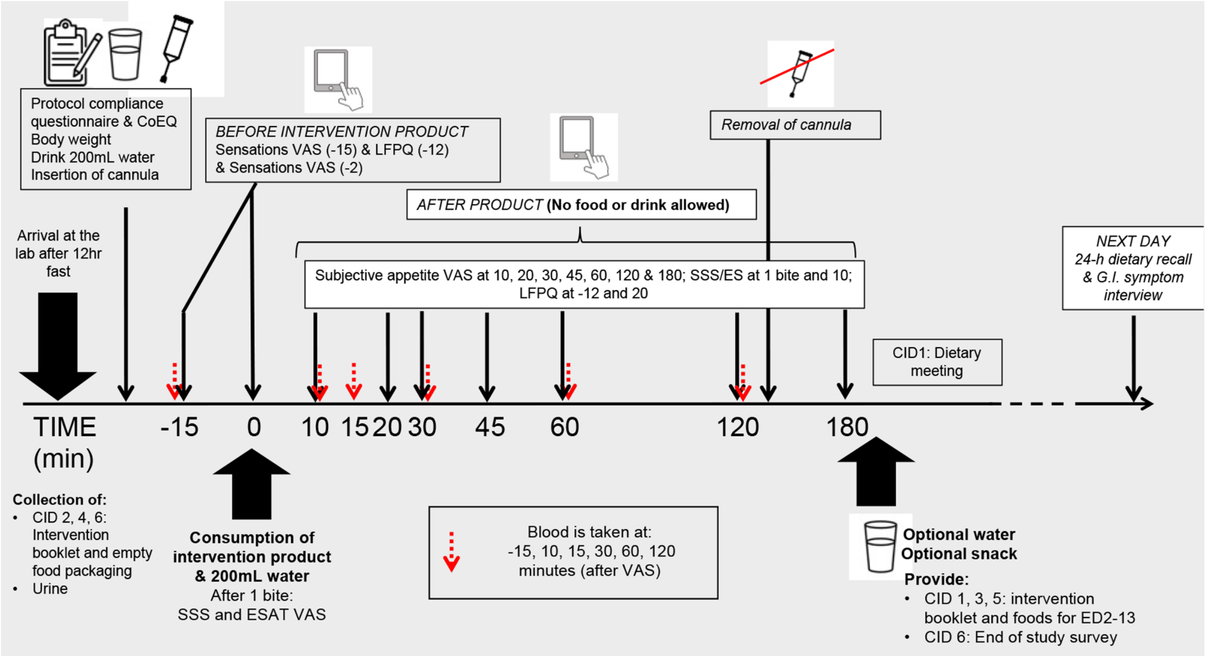

Figure 2 Example timeline of events during clinical investigation day (CID) for biscuit matrix. CoEQ, Control of Eating Questionnaire; ED, exposure day; ESAT, Expected Satiety Questionnaire; GI, gastrointestinal; LFPQ, Leeds Food Preference Questionnaire; SSS, Sensory-Specific Satiety Questionnaire; VAS, Visual Analogue Scale.

desktop. Appetite sensations measured by VAS will be repeated after the LFPQ and before the researcher brings the blinded intervention product served with 200 mL of water. The participant will be instructed to take one bite, then answer questions regarding sensory-specific satiety and expected satiety by VAS.[35 36] The participant will be asked to consume the rest of the product over a period of 5–10 min, depending on the time required to consume the matrix and asked to complete a set of appetite sensation questions by VAS at 10 min, followed by blood samples at 7–10 and 12–15 min to capture peak pancreatic polypeptide (PP) response[37] (yoghurt will be consumed faster than other products; therefore, blood samples will be taken earlier for this matrix). VAS for assessment of appetite sensation will then be taken at 20, 30, 45, 60, 120 and 180 min with blood samples taken after VAS at 30, 60 and 120 min. The LFPQ will be repeated in the fed state after the 20-minute VAS. In between measurements, participants will remain seated in a quiet area, free from food-related sensory stimuli and read/listen to music/use a computer (provided there is no material with reference to food/drink). Once the 180-minute appetite sensation questions by VAS are complete, the participant will be offered water or a snack before leaving the laboratory. Participants will be reminded about the consumption of the products at home and that they will receive a phone call the next day to complete a 24-hour diet recall and report any GI symptoms. Following the end of the trial, participants will be debriefed if requested and offered the chance to complete a survey about the conduct of the study.

Intervention products

There will be one control product (sucrose-containing manufactured products) and two no added/reduced sugar reformulated products based on the same food matrix—including two modulations of S&SE content (inclusion as individual S&SE or S&SE blends). The reformulated products have a target of ≥30% reduction in energy and/or sugar to achieve the status of 'reduced sugar' by EU regulation no 1047/2012. This will not be possible in all products; therefore, 'no added sugar' will be applied to products that do not meet the criteria (biscuits and cakes). The control products will range from 305 to 360 kcal (1286–1516 kJ), while the intervention products will range from 242 to 326 kcal (1013–1368 kJ) (full product nutritional information in online supplemental material 2). Intervention and control products will be matched for sweetness intensity, flavour and physical appearance.

The two individual S&SEs selected based on published literature were Neotame and Stevia Rebaudioside M (in the biscuits and cakes) and two further S&SE blends were Sucralose/Acesulfame K blend and Mogroside V/Stevia Rebaudioside M blend (in yoghurt, chocolate and cereal), selected based on the results of a preliminary study using a beverage matrix (manuscript in preparation).

Data collection and outcomes

Table 1 details at which time point(s) data are collected at the CID.

Primary outcome

This trial has one primary outcome which is the iAUC for the 180-minute composite appetite score based on

BMJ Open: first published as 10.1136/bmjopen-2022-063903 on 23 December 2022. Downloaded from http://bmjopen.bmj.com/ on January 5, 2023 by guest. Protected by copyright.

Table 1 Data collection and time points for each CID

		Baseline or 0’ (fasting)	10’	15’	20’	30’	45’	60’	120’	180’	Next day
Primary endpoint	Subjective appetite (VAS for hunger, desire to eat, fullness, prospective food consumption)	X	X		X	X	X	X	X	X	
Behavioural endpoints	Food preference and reward (LFPQ)	X			X						
	Food cravings (CoEQ)	X									
	Energy intake (24-hour dietary recall)										X
	Expected satiety	X (1 bite)									
	Sensory-specific satiety	X (1 bite)	X								
	Other appetite ratings (eg, thirst, nausea, bloating, appetite for something sweet/savoury)	X	X		X		X	X	X	X	
Metabolic endpoints	Glucose and insulin	X	X	X		X		X	X		
	Pancreatic polypeptide (PP)*	X	X	X		X					
	GLP-1 and ghrelin	X				X		X			
	Lipaemia (triglycerides, total cholesterol, HDL-cholesterol and LDL-cholesterol)	X				X		X	X		
Health endpoints	Liver function (ALT, AST, GGT, FL index, TyG index)	X							X		
	HbA1c	CID1 and 6									
	24-hour GI side effects (self-report)										X

*Time points for PP are earlier for yoghurt study.
ALT, alanine transaminase; AST, aspartate transaminase; CID, clinical investigation day; CoEQ, Control of Eating Questionnaire; FL index, fatty liver index; GGT, gamma-glutamyltransferase; GI, gastrointestinal; GLP-1, glucagon-like peptide 1; HbA1c, haemoglobin A1c; HDL, high-density lipoprotein; LDL, low-density lipoprotein; LFPQ, Leeds Food Preference Questionnaire; TyG index, triglycerides and glucose index; VAS, Visual Analogue Scale.

hunger, fullness (reverse scored), desire to eat and prospective food consumption.[38] These subjective appetite ratings will be measured throughout the CIDs using VAS on the QDP. The trapezoid method will be used for the calculation of iAUC.[26]

Secondary outcomes

Food preference and reward

Food preference and food reward will be measured at all CIDs using the LFPQ.[34] Changes will be determined by comparing the relative preference/food choice, explicit liking and implicit wanting for high-fat sweet, low-fat sweet, high-fat savoury and low-fat savoury foods, and fat/sweet appeal bias scores in the fed and hungry states between the reformulated and control products.

Food cravings

Food cravings will be determined at all CIDs by craving control, craving for sweet and savoury scores from the CoEQ,[31] which is a 21-item questionnaire with responses recorded on a 100-point VAS (one item allows for text response).

Energy intake

Energy intake will be measured by a 24-hour dietary recall (using the multiple pass method[39]), which will be conducted by a trained dietitian or research staff over the telephone. Participants will be asked to recall all food and drink consumed during the 24-hour period since leaving the laboratory. Participants will receive training on reporting food portions using the Australian Health Survey Food Model Booklet[40] or similar culturally adapted resources.

Compensatory eating behaviour will be determined from the analysis of the 24-hour dietary interview data using energy intake calculated with national nutritional software. The following variables will be considered: (1) energy and macronutrient distribution and (2) per cent energy compensation, defined as the adjustment of energy intake provoked by the intervention products[41] (see online supplemental material 3 for further information).

Expected satiety and sensory-specific satiety

Expected satiety will be measured by the Expected Satiety (ESAT) Questionnaire[35 42] and sensory-specific satiety will be measured by the Sensory-Specific Satiety (SSS) Questionnaire[36] after one bite and full consumption (10’) of the product. Responses to both questionnaires are recorded on a 100-point VAS completed on the QDP. ESAT and SSS will be recorded on all CIDs (see online supplemental material 4 for details of each VAS).

BMJ Open: first published as 10.1136/bmjopen-2022-063903 on 23 December 2022. Downloaded from http://bmjopen.bmj.com/ on January 5, 2023 by guest. Protected by copyright.

Other behavioural ratings

Subjective ratings of thirst, nausea, bloating, appetite for sweet and appetite for savoury will be recorded using 100-point VAS on the QDP regularly throughout the CIDs (table 1).

Biochemical measures

Blood for plasma analyses will be centrifuged at 1500 g at 4°C for 10 min immediately after being collected. Blood for serum analyses will be left to clot for 30–60 min before being centrifuged. Whole blood samples for DNA and haemoglobin A1c (HbA1c) will be frozen immediately after collection. Plasma and serum aliquots will be stored at −80°C until shipment for analyses to Bioaitriki Laboratories (central laboratory) in Athens, Greece.

Insulin concentrations will be determined by chemiluminescent microparticle immunoassay (Abbott Laboratories) using an Abbott Alinity i automated immunoassay system. Ghrelin, glucagon-like peptide 1 and PP concentrations will be determined by ELISA, using an open automated ELISA system. HbA1c will be determined by enzymatic assay (Abbott), which consists of two separate concentration measurements: glycated haemoglobin (HbA1c) and total haemoglobin. The two concentrations are used to determine the per cent HbA1c (National Glycohemoglobin Standardization Program (NGSP) units) or the haemoglobin fraction in mmol/mol (Internal Federation of Clinical Chemistry (IFCC) units). Triglycerides will be determined by glycerol phosphate oxidase method (Abbott). Total cholesterol will be determined by enzymatic (oxidase, esterase and peroxidase) analysis (Abbott). Glucose concentrations will be determined by enzymatic (Hexokinase/G-6-PDH) (Abbott) analysis. High-density lipoprotein (HDL)-cholesterol will be determined by an accelerator selective detergent method (Ultra HDL assay, Abbott) and low-density lipoprotein (LDL)-cholesterol by a selective resolution of LDL particles under dye formation method (Direct LDL assay, Abbott). Aspartate transaminase and alanine transaminase will be determined by enzymatic (NADH (without P-5’-P)) assays and gamma-glutamyltransferase by enzymatic, L-gamma glutamyl-3-carboxy-4-nitroanilide substrate (Abbott). All biochemistry parameters will be analysed by an Abbott Alinity c analyser. Fatty liver index and triglyceride glucose index will be calculated according to information provided in online supplemental material 5.

GI side effects

Any reported unusual GI side effects, including abdominal pain/cramps, heartburn, stomach acid/reflux, nausea, vomiting, abdominal rumbling, bloating, belching, excess gas/wind, bowel movements, stool type, etc, during the study will be recorded at the phone call the day after each CID and each day during the at-home intervention in a booklet including the Bristol Stool Form Scale.[43] The GI symptoms check has been based on the validated Gastrointestinal Symptoms Rating Scale tool.[44]

Statistical analysis plan

Per-protocol analysis will include participants who completed all 6 CIDs and had a level of adherence to the product consumption >80%. The main evaluations for this trial will be to investigate differences between the intervention products (two no added/reduced sugar reformulated S&SE products and one sucrose-sweetened control). Where this is not appropriate for some of the secondary outcomes, descriptive analyses will be used to interpret differences. Data will be pooled across the split-site (Leeds and Lyon) study using the biscuit matrix. Data will be presented as means and SD. Outcome variables will be checked for normality and transformed where necessary. To account for any missing data, analyses will be conducted using linear mixed models. Models will compare S&SE product conditions versus sucrose control in a 3 (S&SE1, S&SE2, sucrose control) × 2 (exposure day 1 and exposure day 14) within-subject design. Model parameters will be adjusted to obtain the best model fit. Adjustments for covariates (eg, age, gender, BMI, intervention site, compliance, protocol deviations, adverse events and concomitant medication) will be applied as necessary, for example, in the event that they influence outcomes. Analyses will be reported as both unadjusted and adjusted models. The American Statistical Association’s policy statement on p values[45] advises that all p values from specified statistical models be reported along with point estimates, effect size and CIs to help interpret the compatibility of the data with the study outcomes; therefore, this procedure will be followed. Otherwise, the level of significance will be set at 0.05.

Safety analysis

Information relating to adverse events (including events relating to GI side effects) and concomitant medication will be tabulated and summarised descriptively.

ETHICS AND MONITORING

Each intervention site has obtained ethical approval from their local ethical committee. The following details the specific ethical committees and the reference numbers: University of Leeds School of Psychology (PSC-127, approved 19 November 2020), Comité de Protection des Personnes Nord-Ouest III (2021-42, approved 28 March 2022), Comité de Ética de la Investigación de la Universidad de Navarra (2021.205, approved 7 March 2022), the Ethical Committee, Region H Denmark (application number H-21078447 approved 27 September 2022) and University of Liverpool Central University Research Ethics Committee D (10659, approved 14 April 2022). All study procedures will be conducted in accordance with the Helsinki Declaration and the study protocol has been registered in a public database (ClinicalTrials.gov NCT04633681; online supplemental material 6). To the extent relevant and reasonable International Council for Harmonisation Good Clinical Practice guidelines will be used, and standard operating procedures will be

BMJ Open: first published as 10.1136/bmjopen-2022-063903 on 23 December 2022. Downloaded from http://bmjopen.bmj.com/ on January 5, 2023 by guest. Protected by copyright.

developed to facilitate the same performance and compliance with the protocol in each centre. All personal data are handled confidentially and stored in accordance with applicable law, GDPR and local laws (see online supplemental material 7). All participants will receive written and oral information about the study and only trained study personnel will provide information, monitor and attest signing of the informed consent form. Where required, monitoring of intervention sites will be performed during the study by the University of Navarra depending on local regulations.

Trial status

The COVID-19 pandemic had a large impact on access to infrastructure and services across all intervention centres. For example, research was halted in some institutions or fewer participants could be scheduled per visit (restrictions related to distance and number of social contacts), recruitment of new staff was frozen, new risk assessments were required, ethical review processes were restricted or extremely prolonged because COVID-19-related protocols were prioritised, procurement of supplies, consumables and services was suspended, and information technology and administrative support was restricted. Further, face-to-face clinical work was put under strain. There were also challenges regarding staff and volunteer sickness plus overall volunteer reluctancy to engage in clinical trials affecting the speed of recruitment and testing.

Nevertheless, recruitment opened in May 2021 for the trial at the Leeds and Lyon intervention centres using the biscuit matrix, with last participant last visit completed in June 2022 for Leeds and expected by October 2022 for Lyon. Recruitment for the trial at Lyon using the cake matrix opened in February 2022. The trials at Liverpool and Pamplona started recruiting in Spring 2022, and Copenhagen are still awaiting ethical approval (August 2022).

Author affiliations

[1]School of Psychology, University of Leeds, Leeds, UK
[2]Institute of Population Health, University of Liverpool, Liverpool, UK
[3]Centre for Nutrition Research, University of Navarra, Pamplona, Spain
[4]IdiSNA, Navarra Institute for Health Research, Pamplona, Spain
[5]Food, Consumer Behaviour and Health Research Centre, School of Psychology, University of Surrey, Guildford, UK
[6]Hospices Civils de Lyon, Rhône-Alpes Research Centre for Human Nutrition, Pierre-Benite, France
[7]Hospices Civils de Lyon, French Obesity Research Centre of France, Pierre-Bénite, France
[8]Precision Nutrition Program, IMDEA, Madrid, Spain
[9]Ingredients, Materials and Nutrition, Cargill, Minneapolis, Minnesota, USA
[10]Department of Nutrition, Exercise and Sports, University of Copenhagen, Copenhagen, Denmark
[11]UMR CNRS GEPEA 6144, Oniris, Nantes, France
[12]School of Nutrition and Translational Research in Metabolism, Maastricht University, Maastricht, The Netherlands
[13]Division of Human Nutrition and Health, Wageningen University, Wageningen, The Netherlands
[14]International Reference Laboratory Services, Bioaitriki, Athens, Greece
[15]Clinical Research, Steno Diabetes Center Copenhagen, Herlev, Denmark

Contributors The SWEET EU Project was initiated by JAH, JCGH and AR. The protocol for the present SWEET work package intervention trial was written by CG, CH, EA-R, SN-C, CEH, JAN, JAM, CS, EEB, EF, HM, KB and GF, with all contributing to the design of the trial along with BO'H, DO'C, MW, MA, MN and CR. JAM, GF, JAN, AR and CH are principal investigators (PI) at the five intervention sites. CG and BO'H drafted the manuscript, and EA-R, SN-C, MW, CS, LK, AR, KB and GF critically reviewed the manuscript. All authors read and approved the final manuscript. Responsible author is CG.

Funding The present study is funded by the Horizon 2020 programme: Sweeteners and sweetness enhancers: Impact on health, obesity, safety and sustainability (acronym: SWEET, grant no: 774293). The current study was initiated by GF as part of the Work Package 2 of the SWEET Project. The study receives funding from the Horizon 2020 programme (€9 million) to cover salary for project personnel, supplies, remuneration and dissemination of results. The amount is deposited in a project account subject to public revision.

Disclaimer The funder has no role in the study design, interpretation of data or publication of material.

Competing interests JCGH, JAH and CH are in receipt of research funding from the American Beverage Association. CH has received honoraria from the International Sweeteners Association. AR has received honoraria from Unilever and the International Sweeteners Association. CEH's research centre provides consultancy to and has received travel funds to present research results from organisations supported by food and drink companies. CS is a paid employee of Cargill.

Patient and public involvement Patients and/or the public were involved in the design, or conduct, or reporting, or dissemination plans of this research. Refer to the Methods section for further details.

Patient consent for publication Not required.

Provenance and peer review Not commissioned; externally peer reviewed.

Supplemental material This content has been supplied by the author(s). It has not been vetted by BMJ Publishing Group Limited (BMJ) and may not have been peer-reviewed. Any opinions or recommendations discussed are solely those of the author(s) and are not endorsed by BMJ. BMJ disclaims all liability and responsibility arising from any reliance placed on the content. Where the content includes any translated material, BMJ does not warrant the accuracy and reliability of the translations (including but not limited to local regulations, clinical guidelines, terminology, drug names and drug dosages), and is not responsible for any error and/or omissions arising from translation and adaptation or otherwise.

Open access This is an open access article distributed in accordance with the Creative Commons Attribution Non Commercial (CC BY-NC 4.0) license, which permits others to distribute, remix, adapt, build upon this work non-commercially, and license their derivative works on different terms, provided the original work is properly cited, appropriate credit is given, any changes made indicated, and the use is non-commercial. See: http://creativecommons.org/licenses/by-nc/4.0/.

ORCID iDs

Catherine Gibbons http://orcid.org/0000-0002-7569-3417
Beverley O'Hara http://orcid.org/0000-0002-4048-9782
Santiago Navas-Carretero http://orcid.org/0000-0002-5163-2230
Charo E Hodgkins http://orcid.org/0000-0003-4775-0338
Louise Kjølbæk http://orcid.org/0000-0003-4310-9332
Ellen E Blaak http://orcid.org/0000-0002-2496-3464
Anne Raben http://orcid.org/0000-0001-5229-4491
Kristine Beaulieu http://orcid.org/0000-0001-8926-6953
Graham Finlayson http://orcid.org/0000-0002-5620-2256

REFERENCES

1 Mitchell NS, Catenacci VA, Wyatt HR, *et al*. Obesity: overview of an epidemic. *Psychiatr Clin North Am* 2011;34:717–32.
2 Organisation WH. *Guideline: sugars intake for adults and children*. Geneva: World Health Organisation, 2015.
3 San-Cristobal R, Navas-Carretero S, Martínez-González Miguel Ángel, *et al*. Contribution of macronutrients to obesity: implications for precision nutrition. *Nat Rev Endocrinol* 2020;16:305–20.
4 Anguah KO-B, Syed-Abdul MM, Hu Q, *et al*. Changes in food cravings and eating behavior after a dietary carbohydrate restriction intervention trial. *Nutrients* 2019;12. doi:10.3390/nu12010052. [Epub ahead of print: 24 Dec 2019].

BMJ Open: first published as 10.1136/bmjopen-2022-063903 on 23 December 2022. Downloaded from http://bmjopen.bmj.com/ on January 5, 2023 by guest. Protected by copyright.

5 Zellner DA, Garriga-Trillo A, Rohm E, *et al*. Food liking and craving: a cross-cultural approach. *Appetite* 1999;33:61–70.
6 O'Connor D, Pang M, Castelnuovo G, *et al*. A rational review on the effects of sweeteners and sweetness enhancers on appetite, food reward and metabolic/adiposity outcomes in adults. *Food Funct* 2021;12:442–65.
7 Hunter SR, Reister EJ, Cheon E, *et al*. Low calorie sweeteners differ in their physiological effects in humans. *Nutrients* 2019;11:2717.
8 Pang MD, Goossens GH, Blaak EE. The impact of artificial sweeteners on body weight control and glucose homeostasis. *Front Nutr* 2020;7:598340.
9 Higgins KA, Mattes RD. A randomized controlled trial contrasting the effects of 4 low-calorie sweeteners and sucrose on body weight in adults with overweight or obesity. *Am J Clin Nutr* 2019;109:1288–301.
10 Lee HY, Jack M, Poon T, *et al*. Effects of unsweetened preloads and preloads sweetened with caloric or low-/no-calorie sweeteners on subsequent energy intakes: a systematic review and meta-analysis of controlled human intervention studies. *Adv Nutr* 2021;12:1481–99.
11 Rogers PJ, Ferriday D, Irani B, *et al*. Sweet satiation: acute effects of consumption of sweet drinks on appetite for and intake of sweet and non-sweet foods. *Appetite* 2020;149:104631.
12 Stamataki NS, Crooks B, Ahmed A, *et al*. Effects of the daily consumption of stevia on glucose homeostasis, body weight, and energy intake: a randomised open-label 12-week trial in healthy adults. *Nutrients* 2020;12. doi:10.3390/nu12103049. [Epub ahead of print: 06 Oct 2020].
13 Magnuson BA, Carakostas MC, Moore NH, *et al*. Biological fate of low-calorie sweeteners. *Nutr Rev* 2016;74:670–89.
14 Swithers SE. Artificial sweeteners produce the counterintuitive effect of inducing metabolic derangements. *Trends Endocrinol Metab* 2013;24:431–41.
15 McGlynn ND, Khan TA, Wang L, *et al*. Association of low- and no-calorie sweetened beverages as a replacement for sugar-sweetened beverages with body weight and cardiometabolic risk: a systematic review and meta-analysis. *JAMA Netw Open* 2022;5:e222092.
16 Nunn R, Young L, Ni Mhurchu C. Prevalence and types of non-nutritive sweeteners in the New Zealand food supply, 2013 and 2019. *Nutrients* 2021;13. doi:10.3390/nu13093228. [Epub ahead of print: 16 Sep 2021].
17 Ng SW, Colchero MA, White M. How should we evaluate sweetened beverage tax policies? a review of worldwide experience. *BMC Public Health* 2021;21:1941.
18 Russell C, Grimes C, Baker P, *et al*. The drivers, trends and dietary impacts of non-nutritive sweeteners in the food supply: a narrative review. *Nutr Res Rev* 2021;34:185–208.
19 Webster J. *Working paper on product reformulation and portion size*. Brussels: EU Platform on diet, physical activity and health, 2009.
20 Organisation WH. Sugar-Sweetened beverage taxes in the who European region: success through lessons learned and challenges faced, who regional office for Europe Licence: CC BY-NC-SA 3.0 IGO, contract NO: WHO/EURO:2022-4781-44544-6381. Copenhagen; 2022.
21 Rogers PJ, Hogenkamp PS, de Graaf C, *et al*. Does low-energy sweetener consumption affect energy intake and body weight? A systematic review, including meta-analyses, of the evidence from human and animal studies. *Int J Obes* 2016;40:381–94.
22 Chan A-W, Tetzlaff JM, Altman DG, *et al*. Spirit 2013 statement: defining standard protocol items for clinical trials. *Ann Intern Med* 2013;158:200–7.
23 Kjølbæk L, Manios Y, Blaak EE, *et al*. Protocol for a multicentre, parallel, randomised, controlled trial on the effect of sweeteners and sweetness enhancers on health, obesity and safety in overweight adults and children: the sweet project. *BMJ Open* 2022;12:e061075.
24 Almiron-Roig E, Green H, Virgili R, *et al*. Validation of a new hand-held electronic appetite rating system against the pen and paper method. *Appetite* 2009;53:465–8.
25 Jones B, Kenward MG. *Design and analysis of cross-over trials*. 3rd Edition. Boca Raton, Florida: CRC Press, 2015.
26 Blundell J, de Graaf C, Hulshof T, *et al*. Appetite control: methodological aspects of the evaluation of foods. *Obes Rev* 2010;11:251–70.
27 Yeomans MR, Re R, Wickham M, *et al*. Beyond expectations: the physiological basis of sensory enhancement of satiety. *Int J Obes* 2016;40:1693–8.
28 Garner DM, Garfinkel PE. The eating attitudes test: an index of the symptoms of anorexia nervosa. *Psychol Med* 1979;9:273–9.
29 Masic U, Harrold JA, Christiansen P, *et al*. Effects of non-nutritive sWeetened beverages on appetIte during active weigHt loss (switch): protocol for a randomized, controlled trial assessing the effects of non-nutritive sWeetened beverages compared to water during a 12-week weigHt loss period and a follow up weight maintenance period. *Contemp Clin Trials* 2017;53:80–8.
30 Booth M. Assessment of physical activity: an international perspective. *Res Q Exerc Sport* 2000;71 Suppl 2:114–20.
31 Dalton M, Finlayson G, Hill A, *et al*. Preliminary validation and principal components analysis of the control of eating questionnaire (CoEQ) for the experience of food craving. *Eur J Clin Nutr* 2015;69:1313–7.
32 Brunger L, Smith A, Re R, *et al*. Validation of an iPad visual analogue rating system for assessing appetite and satiety. *Appetite* 2015;84:259–63.
33 Gibbons C, Caudwell P, Finlayson G, *et al*. Validation of a new hand-held electronic data capture method for continuous monitoring of subjective appetite sensations. *Int J Behav Nutr Phys Act* 2011;8:57.
34 Finlayson G, King N, Blundell J. The role of implicit wanting in relation to explicit liking and wanting for food: implications for appetite control. *Appetite* 2008;50:120–7.
35 Forde CG, Almiron-Roig E, Brunstrom JM. Expected satiety: application to weight management and understanding energy selection in humans. *Curr Obes Rep* 2015;4:131–40.
36 Snoek HM, Huntjens L, Van Gemert LJ, *et al*. Sensory-specific satiety in obese and normal-weight women. *Am J Clin Nutr* 2004;80:823–31.
37 Lasschuijt MP, Mars M, de Graaf C, *et al*. Endocrine cephalic phase responses to food cues: a systematic review. *Adv Nutr* 2020;11:1364–83.
38 Anderson GH, Catherine NLA, Woodend DM, *et al*. Inverse association between the effect of carbohydrates on blood glucose and subsequent short-term food intake in young men. *Am J Clin Nutr* 2002;76:1023–30.
39 Steinfeldt L, Anand J, Murayi T. Food reporting patterns in the USDA automated multiple-pass method. *Procedia Food Sci* 2013;2:145–56.
40 Australian Health Survey. Food Model Booklet. In: ABo S, ed. Belconnen ACT, 2010.
41 Zandstra EH, Mathey MF, Graaf C, *et al*. Short-term regulation of food intake in children, young adults and the elderly. *Eur J Clin Nutr* 2000;54:239–46.
42 Brunstrom JM, Shakeshaft NG, Scott-Samuel NE. Measuring 'expected satiety' in a range of common foods using a method of constant stimuli. *Appetite* 2008;51:604–14.
43 O'Donnell LJ, Virjee J, Heaton KW. Detection of pseudodiarrhoea by simple clinical assessment of intestinal transit rate. *BMJ* 1990;300:439–40.
44 Svedlund J, Sjödin I, Dotevall G. GSRS--a clinical rating scale for gastrointestinal symptoms in patients with irritable bowel syndrome and peptic ulcer disease. *Dig Dis Sci* 1988;33:129–34.
45 Wasserstein RL, Lazar NA. The ASA Statement on p -values: context, process, and purpose. *Am Stat* 2016;70:129–33.

BMJ Open: first published as 10.1136/bmjopen-2022-063903 on 23 December 2022. Downloaded from http://bmjopen.bmj.com/ on January 5, 2023 by guest. Protected by copyright.
