# Supplementary material for: Acute and two-week effects of neotame, stevia rebaudioside M and sucrose-sweetened biscuits on postprandial appetite and endocrine response in adults with overweight/obesity—a randomised crossover trial from the SWEET consortium
Source: eBioMedicine. 2024 Mar 28;102:105005. doi: 10.1016/j.ebiom.2024.105005 (PMC11026940; doi:10.1016/j.ebiom.2024.105005)
Supplement: List of consortia members [file mmc3.docx]

|  |  |  |
| --- | --- | --- |
|  |  |  |
|  | **First Names** | **Surnames** |
|  | Charlotte | Hardman |
|  | Moon | Wilton |
|  | Paul | Christiansen |
|  | Eva | Almiron-Roig |
|  | Alfredo | Martinez-Hernandez |
|  | Santiago | Navas-Carretero |
|  | Scott | Corey |
|  | Edith | Feskens |
|  | Naomi | Novita |
|  | Elske | Brouwer-Brolsma |
|  | Marion | Buso |
|  | Adam | Tanja |
|  | Michelle | Pang |
|  | Gijs | Goossens |
|  | Charo | Hodgkins |
|  | Stephen | Morse |
|  | Monique | Raats |
|  | Maria | Flourakis |
|  | Ching-Yu | Chang |
|  | Jasper | Scholten |
|  | Heleen | Claringbould |
|  | Graham | Finlayson |
|  | Catherine | Gibbons |
|  | Kristine | Beaulieu |
|  | Lisa | Heggie |
|  | Tony | Lam |
|  | Torsten | Voegler |
|  | George | Nychas |
|  | Sabine | Klager |
|  | Marta | Delalomo |
|  | Hariklia | Moshoyianni |
|  | Abel | Crespo |
|  | Ascensia | Ciruelos |
|  | alain | Le-Bail |
|  | Mathilde | Roze |
|  | Nicolas | Metzger |
|  | Loic | Briand |
|  | Christine | Belloir |
|  | Sian | Astley |
|  | Mark | Roe |
|  | Paul | Finglas |
|  | Sharron | Kuznesof |
|  | Eleni | Hadjigeorgiou |
|  | Maud | Alligier |
|  | Laurie | Vandenberghe |
|  | Eleni | Triantafyllou |
|  | Kyriakos | Reppas |
|  | Eirini | botsi |
|  | Kiriaki | Apergi |
|  | Claire | Llewellyn |
|  | Richard | Murphy |
|  | Lada | Timotijevic |
|  | Julie-Anne | Nazare |
|  | Hana | Musinovic |
|  | James | Suckling |
|  | Ellen | Blaak |
|  | Anne | Raben |
|  | Sabina | Andersen |
|  | Euan | Woodward |
|  | Sheree | Bryant |
|  | Louise | Kjoelbaek |
|  | Yannis | Manios |
|  | Jacco | Bastings |
|  | Maria-Isabel | Cisneros |
|  | Giorgia | Sabbatini |
|  | Jason | Halford |
|  | Clarissa | Dakin |
|  | Dominic | O'Connor |
|  | Beverley | O'Hara |
